# Cathepsin g Degrades Both Glycosylated and Unglycosylated Regions of Lubricin, a Synovial Mucin

**DOI:** 10.1038/s41598-020-61161-5

**Published:** 2020-03-06

**Authors:** Shan Huang, Kristina A. Thomsson, Chunsheng Jin, Sally Alweddi, André Struglics, Ola Rolfson, Lena I. Björkman, Sebastian Kalamajski, Tannin A. Schmidt, Gregory D. Jay, Roman Krawetz, Niclas G. Karlsson, Thomas Eisler

**Affiliations:** 1 Department of Medical Biochemistry and Cell Biology, Institute of Biomedicine, Sahlgrenska Academy, University of Gothenburg, Gothenburg, Sweden; 2 Science for Life Laboratory, Department of Oncology-Pathology, Clinical Proteomics Mass Spectrometry, Karolinska Institutet, Solna, Sweden; 3 Department of Clinical Sciences Lund, Orthopaedics, Faculty of Medicine, Lund University, Lund, Sweden; 4 Department of Orthopaedics, Institute of Clinical Sciences, The Sahlgrenska Academy, University of Gothenburg, Gothenburg, Sweden; 5 Department of Rheumatology and Inflammation Research, Institute of Medicine, Sahlgrenska Academy, University of Gothenburg, Gothenburg, Sweden; 6 Department of Molecular Skeletal Biology, Lund University, Lund, Sweden; 7 Biomedical Engineering Department, University of Connecticut Health Centre, Farmington, CT USA; 8 Department of Emergency Medicine, Warren Alpert Medical School and Division of Biomedical Engineering, School of Engineering, Brown University, Providence, RI USA; 9 Cell Biology and Anatomy, Cumming School of Medicine, University of Calgary, 3330 Hospital Drive NW, Calgary, Alberta T2N4N1 Canada; 10 McCaig institute for Bone and Joint Health, University of Calgary, Calgary, Alberta T2N4N1 Canada; 11 Department of Clinical Sciences, Danderyd Hospital, Karolinska Institutet, Stockholm, Sweden

**Keywords:** Biochemistry, Structural biology, Biomarkers, Medical research, Rheumatology

## Abstract

Lubricin (PRG4) is a mucin type protein that plays an important role in maintaining normal joint function by providing lubrication and chondroprotection. Improper lubricin modification and degradation has been observed in idiopathic osteoarthritis (OA), while the detailed mechanism still remains unknown. We hypothesized that the protease cathepsin G (CG) may participate in degrading lubricin in synovial fluid (SF). The presence of endogenous CG in SF was confirmed in 16 patients with knee OA. Recombinant human lubricin (rhPRG4) and native lubricin purified from the SF of patients were incubated with exogenous CG and lubricin degradation was monitored using western blot, staining by Coomassie or Periodic Acid-Schiff base in gels, and with proteomics. Full length lubricin (∼300 kDa), was efficiently digested with CG generating a 25-kDa protein fragment, originating from the densely glycosylated mucin domain (∼250 kDa). The 25-kDa fragment was present in the SF from OA patients, and the amount was increased after incubation with CG. A CG digest of rhPRG4 revealed 135 peptides and 72 glycopeptides, and confirmed that the protease could cleave in all domains of lubricin, including the mucin domain. Our results suggest that synovial CG may take part in the degradation of lubricin, which could affect the pathological decrease of the lubrication in degenerative joint disease.

## Introduction

Lubricin, also known as Proteoglycan 4 (PRG4) is a large (∼300 kDa) extensively *O*-linked glycosylated mucinous protein in SF that plays a critical role in maintaining cartilage integrity by providing boundary lubrication and reducing friction at the cartilage surface^1^. Besides the principle function of lubrication, lubricin also has growth-regulating properties^2^, prevents cell adhesion, provides chondroprotection^3–5^, and plays a role in the maturation of the subchondral bone^6^. Lubricin is predominantly synthesized and expressed by superficial zone chondrocytes at the cartilage surface layer^7^, but can also be secreted into the SF by synovial fibroblasts^8^ and stromal cells from peri-articular adipose tissues^9^. More recently it was reported that other sites and tissues, such as tendons, liver, kidney, skeletal muscles, and the ocular surface, also express lubricin^10–12^.

**Figure 1:**
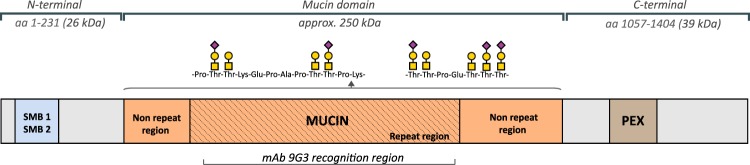
(**a**) Description of lubricin and its domains. Lubricin contains a heavily glycosylated Pro/Thr rich mucin domain, flanked by two somatomedin B domains (SMB) and a hemopexin (PEX) domain. The *O*-glycans are linked to Thr and Ser residues, and increases the size of the mucin domain from 86 kD (without glycans) to approximately 250 kD. The monoclonal lubricin antibody 9G3 has been shown to bind the glycosylated peptide sequence ‘KEPAPTTT’ which is found eight times in the repeat region. Monosaccharides constituting lubricin oligosaccharides are represented by Symbol Nomenclature for Glycans (SNFG)^57^, where *N*-acetylgalactosamine (GalNAc) is a yellow square, galactose (Gal) is a yellow circle and *N*-acetylneuraminic acid (NeuAc) is a purple diamond.

In the joint, lubricin is an extended molecule existing both as monomers and as higher molecular mass complexes^11^. Lubricin has an approximately equal mass proportion of protein and oligosaccharides, and contains one central mucin-like region, two somatomedin B homology domains (SMB) in the *N*-terminal, one heparin or chondroitin sulfate binding domain, and one hemopexin-like domain (PEX)^10,13^ in the *C*-terminal (Fig. 1). The multiple domain structure contributes to diverse biological roles of lubricin. The *O*-linked oligosaccharides in the mucin domain render lubricin a low friction brush-like construct with repulsive hydration forces, provide lubrication during boundary movement, and makes lubricin water-soluble in SF^14^. The oligosaccharides are mostly linked to abundant Thr residues, which are found in the repeat region, consisting of repeats with minor variations of the amino acid sequence ‘EPAPTTPK’, as well as its flanking, non-repeating Thr/Ser rich regions. The sparingly glycosylated terminal regions SMB and PEX domains have in other proteins been demonstrated to regulate innate immune processes by interacting with both complement and coagulation factors^15^ and assisting matrix protein binding^16^.

For over a decade, researchers have demonstrated that lubricin plays a vital role in inflammatory joint disease. Prevention of lubricin degradation may serve as a therapy in an early stage inflammatory arthritis^17–21^. Altered lubricin structure and change of lubricin concentrations, which disrupts cartilage boundary lubrication, is found in SF of Osteoarthritis (OA) patients^22^. As such, lubricin degradation may play a role in OA associated inflammation. OA is the most common degenerative joint disease, which affects people worldwide^23^. The disease involves a progressive destruction of cartilage, bone and ligaments, sometimes causing extensive pain, reduced joint flexibility and may lead to severely impaired quality of life and rising health care costs. Recent research emphasizes idiopathic OA as a multifactorial joint disease^24^ with an increasing number of studies demonstrating a low-grade inflammation and an accumulation of pro-inflammatory cytokines in synovial fluid (SF), which is associated with the pathological process of OA^25^. Cytokine triggered enzymatic degradation of matrix proteins is believed to contribute to the cartilage erosion found in OA^26–29^.

A limited number of proteases have been identified to degrade lubricin, among the more studied are lysosomal cysteine proteases cathepsin B, S, L and neutrophil elastase^19,21,30^. Cathepsin G (CG) is one of the major neutrophil serine proteases that is synthesized in bone marrow and subsequently stored in the azurophil granules of polymorphonuclear neutrophils^31,32^. CG exhibit optimal activity in a broad pH range (pH 7–8)^33^, operational in the pH level reported for SF is (pH 7.5–7.78)^34^. Upon activation of the granulocytes, CG is released at sites of inflammation and plays a crucial part in degrading chemokines and extracellular matrix (ECM) proteins, as well as regulating and activating pro-inflammatory cytokines^35^. Therefore, CG is reported to be active in various chronic inflammatory diseases^36^ and has for instance been shown to be highly active in the SF of rheumatoid arthritis patients. CG has also been reported to be present in OA synovial fluid^37^ and synovial lining^38^.

Despite evidence indicating that lubricin modification contributes to some to OA initiation and pathological development, research and understanding of enzymatic degradation of lubricin in OA is still limited. Our data confirmed previous reports of endogenous CG in OA, by detection of the 29 kDa protease in a panel of SF samples from 16 patients with idiopathic knee OA. This initiated the investigation of the ability of CG to degrade lubricin. A 25 kDa lubricin fragment constituting part of its mucin domain was identified in these patients. The relative abundance of this fragment was increased after incubation of exogenous CG added to OA SF. In addition, CG generated fragments of recombinant lubricin were identified using proteomics.

## Results

### Detection of a synovial CG: western blot confirmation of CG in synovial fluids of OA subjects

**Figure 2:**
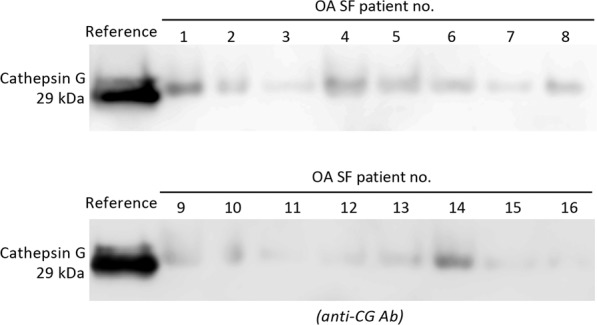
Endogenous Cathepsin G (CG) in synovial fluid (SF) from OA patients. CG was detected in SF from 16 OA patients. SF was analyzed with SDS-PAGE followed by western blot using a polyclonal anti-CG antibody. Bands corresponding to CG were detected at 29 kDa, and compared to a reference compound analyzed on the same gel (22 ng CG). The full-length western blots are displayed in Supplementary Fig. S1.

CG has previously been shown to be present in OA SF, however in a low abundance compared to SF from rheumatoid arthritis patients^37^. We verified in our SF sample collection that CG was present in OA. SF samples were collected from 16 late-stage OA patients and analyzed for the presence of CG using SDS-PAGE and western blot with a polyclonal anti-CG antibody. We could detect CG in all the SF samples (Fig. 2). By including 22 ng/μL CG in a separate lane as a positive control, it was obvious that the amount of CG in the SF samples was in line with a previous study where a concentration of 1–5 ng/μL CG in OA SF was reported^37^. This data inspired us to further investigate the connection between lubricin and CG degradation.

### CG degrades recombinant and native lubricin

**Figure 3:**
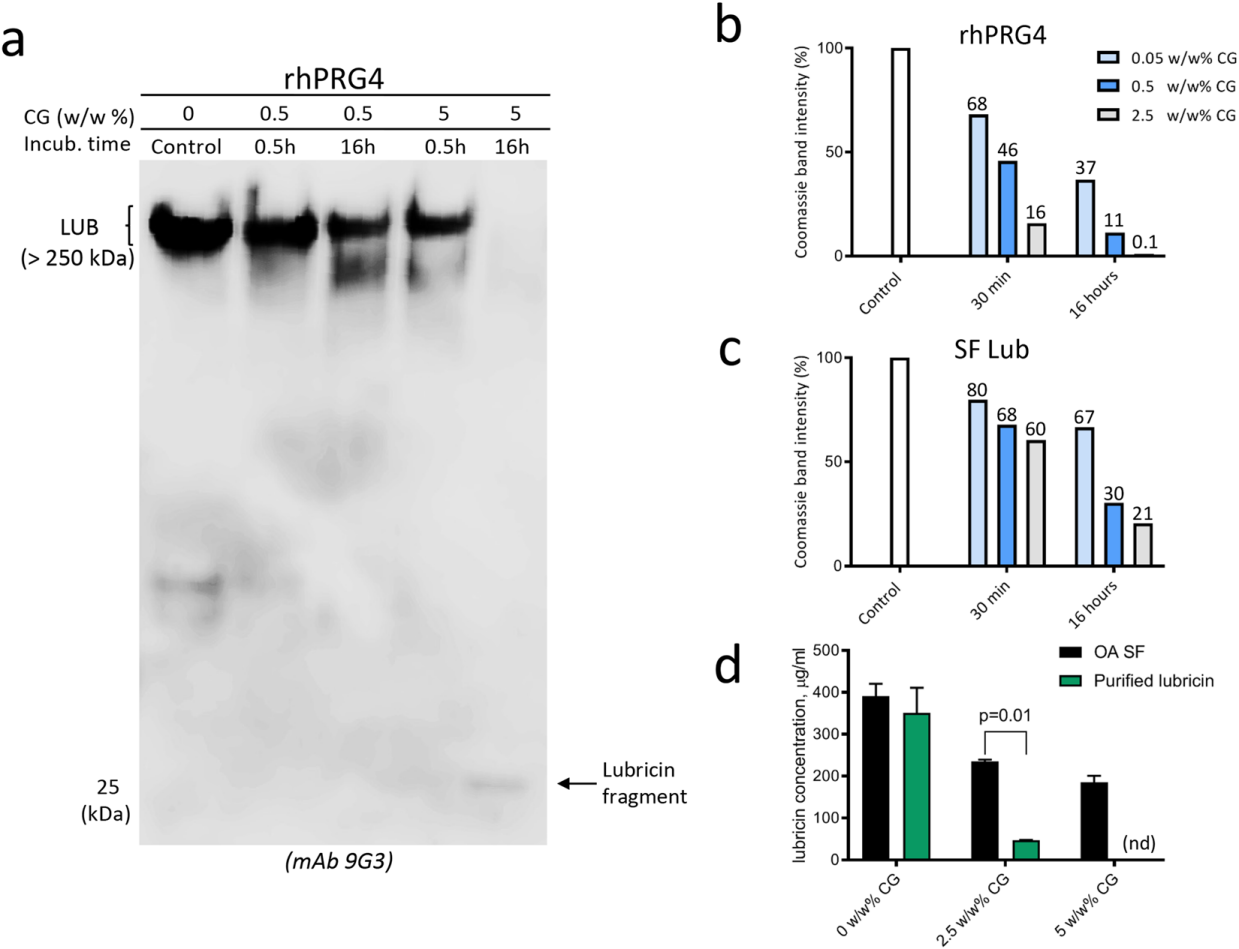
Cathepsin G (CG) degrades lubricin. rhPRG4 and purified lubricin from synovial fluid (SF Lub) were incubated with CG or without CG (control) at increasing enzyme to substrate ratios (weight/weight) in PBS for 30 min or 16 hours, followed by analyses on tris-acetate gels (3–8%). rhPRG4 was detected with western blot using mAb 9G3 **(a)** or using Coomassie Blue stain (rhPRG4 and purified SF lubricin. gels from repeated experiments are displayed in Supplementary Figs S2 and S3). Coomassie Blue band intensities from a typical experiment of full length lubricin incubated with CG compared to the control lubricin band (no CG) for one gel are displayed in **(b**,**c)** with the relative intensities in % displayed as a number above each bar to numerically display difference in digestion comparing rhPRG4 and native lubricin. **(d)** CG degradation of native lubricin is decreased in synovial fluid compared to in PBS. SF (5 μl) from an OA patient and purified SF lubricin in PBS from SF (2.5 μg in 5 μL PBS) were incubated with CG in PBS at 37 °C for two hours. Lubricin was detected using a sandwich ELISA, with mAb 9G3 as the catching Ab and PNA lectin for detection (see Materials and Methods section). Samples were measured in duplicates and mean +/− SEM are shown. Significant differences were calculated by ordinary two-way ANOVA with Holm-Sidak’s multiple comparision.

In order to investigate if CG was able to digest lubricin, we performed incubations using recombinant lubricin (rhPRG4). The full length glycosylated protein and its degradation products were separated with SDS-PAGE and detected with lubricin mAb 9G3. This antibody targets part of the glycosylated mucin domain^12^ (Fig. 1). The results indicated that rhPRG4 was indeed degraded after extended incubation, by observing the decreased antibody staining intensity of the full length protein in conjunction with a major degradation product detected at approximately 25 kDa (Fig. 3a).

To compare differences in the ability of CG to digest different forms of lubricin, we monitored the degradation using rhPRG4 and native lubricin purified from patients using a Coomassie protein staining. Coomassie was chosen rather than mAb 9G3 western blot, since we initially wanted to monitor CG degradation of the unglycosylated *N-* and *C*-terminal regions, hypothesizing that they were its primary targets. Both rhPRG4 and native lubricin purified from SF were found to be degraded in a dose-dependent manner. The degradation of rhPRG4 and native lubricin was found to increase with prolonged incubation time (Fig. 3b,c and Supplementary Figs. S2 and S3). No major bands of lower molecular mass peptide products were detected in the Coomassie stained SDS-PAGE gels in neither of the lubricin forms. This indicated that most generated non-glycosylated peptides were of low molecular mass (<10 KDa) below the SDS-PAGE range. However, the two lubricin forms were found to be degraded at different rates. rhPRG4 was found to be efficiently degraded by CG already after 30 minutes of incubation. This was illustrated by a semiquantitative measurement using the Coomassie stain, showing in one of the experiment that 32%, 54% and 84% of the full length protein had been degraded with increasing CG-to-protein ratio, respectively. In this experiment, rhPRG4 was completely degraded after 16 hours (0.1% left) (Fig. 3b) using the highest concentration of CG (2.5 w/w %CG). Native lubricin was found to be more resilient to CG digestion. After 16 hours of incubation with the higher CG concentrations (0.5 and 2.5 w/w % CG), 30 and 21% of the full length lubricin remained undigested, respectively. (Fig. 3c). Similar difference between the susceptibility of rhPRG4 and native lubricin to CG degradation was found in repeat experiments (Supplementary Figs. S2 and S3).

These results suggested that the protein core of native lubricin was less accessible for CG digestion. However, while the bulk of natural SF protease inhibitors are removed using ion exchange chromatography in the purification of native lubricin, proteomic analysis indicated that low level still remained^39^. In order to monitor the effect of endogenous CF proteases, we measured the difference between native lubricin degradation by supplementing SF (high level of proteases inhibitor) with exogenous CG and compared to when CG was added to purified native lubricin (protease inhibitor depleted) in PBS. For this we adopted a sandwich ELISA (Materials and Methods) to be able to monitor lubricin in presence of other proteins. With this ELISA we could show that the high level of protease inhibitors present in SF significantly influenced the degradation of lubricin (Fig. 3d). Finally, to confirm that lubricin degradation was due to CG, the degradation of purified SF lubricin was shown to be totally abolished using CG inhibitors (Supplementary Fig. S4). In all, we concluded that CG was capable of degrading lubricin both in its recombinant and native form and that natural SF proteases inhibitor inhibited the CG activity.

### CG degrades the lubricin mucin domain

**Figure 4:**
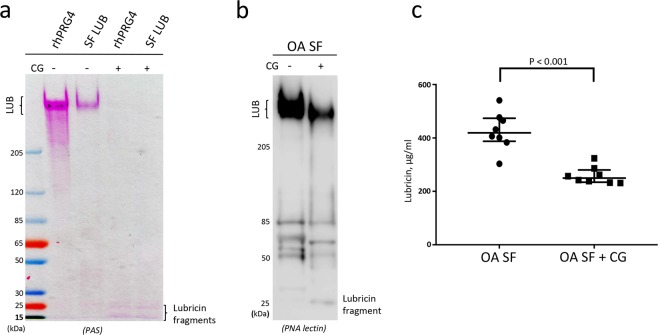
Periodic Acid Schiff (PAS) and Peanut Agglutinin (PNA) lectin detection of lubricin and glycosylated lubricin fragments after incubation with Cathepsin G (CG). **(a)** PAS glycostaining of recombinant lubricin (rhPRG4) and purified lubricin from synovial fluid (SF LUB). rhPRG4 and SF LUB were incubated with 5 w/w% CG (enzyme to substrate ratio by weight) in PBS for 2 hours at 37 °C, followed by analysis with SDS-PAGE and staining with PAS. **(b)** PNA lectin western blot oSF incubated with CG. SF (2.5 μL) from an OA patient was incubated with or without CG (55 ng) for 2 hours at 37 °C. PNA stains mucin *O*-glycans (Galβ1-3GalNAc-). **(c)** Lubricin quantification in SF using ELISA. SF (2 μL) from eight OA patients were incubated with CG (44 ng) or without CG for 2 hours at 37 °C. Lubricin was measured using a sandwich ELISA, with mAb 9G3 as the catching Ab and *Peanut agglutinin* (PNA) lectin for detection, and compared to a standard curve with rhPRG4. Samples were measured in duplicates and mean values are presented as mean +/− SEM. Significant differences were calculated by Mann-Whitney test.

The decrease in Coomassie stain of lubricin after incubation with CG (Fig. 3b,c and Supplementary Figs. S2 and S3) indicated that the unglycosylated protein parts was substantially effected. In addition, using mAb 9G3 based detection in western blot (Fig. 3a) and ELISA (Fig. 3d), indicated that CG was a potent protease also capable of attacking the lubricin glycosylated mucin domain. Mucin domains are renowned for being hard to digest with proteolytic enzymes. In order to monitor how the whole mucin domain was affected by CG degradation and not only the region recognized by mAb 9G3, we adopted the carbohydrate sensitive Periodic Acid Schiff’s base (PAS) stain after CG degradation of lubricin and SDS-PAGE separation of the proteolytic products. Recombinant (rhPRG4) and purified lubricin from SF were incubated for two hours with CG, followed by separation on gels and PAS staining. This general glycostain reacts with the all *O*-glycans present on lubricin. It visualized the presence of a heavily *O*-glycosylated intact mucin domains shown by the intense staining before CG digestion of rhPRG4 and native lubricin. After digestion only two faintly stained glycosylated degradation products (15- and 25-kDa) were detected on the SDS-PAGE gels (Fig. 4a), both for rhPRG4 and native lubricin. This indicated that most glycopeptides generated after incubation remained undetected using SDS-PAGE and that additional proteolytic glycosylated peptides may be present as lower mass. The result supported previous indication in this report, that both the glycosylated and non-glycosylated regions of lubricin were proteolytically digested by CG.

We also then examined CG degradation of the lubricin mucin domain in SF where the natural SF protease CG inhibitors were shown to be present as described above. Due to presence of many different glycoproteins in SF, the western blot was developed using peanut agglutinin (PNA) lectin. PNA binds mucin type *O*-glycans (Galβ1–3GalNAc-) that are abundant on lubricin mucin domain^10^. Using this approach we detected full length lubricin in SF (Fig. 4A). After two hours of incubation of OA SF with added CG, the intensity of the band corresponding to full length lubricin had decreased considerably compared to the control without CG, and the formation of a weakly stained band at 25 kDa was observed (Fig. 4b) consistent with previous results. Western blot using PNA of SF proteins incubated without CG revealed that in addition to full length lubricin, several other PNA reactive components of unknown origin at 50–100 kDa was detected. These components probably represent other PNA reactive proteins in SF as well as degradation products of lubricin.

Using sandwich ELISA we also quantitatively assessed the ability of CG to digest native lubricin present in SF. Eight OA SF samples were incubated with or without supplement of exogenous CG. Lubricin was quantified using ELISA with mAb 9G3 as the catching antibody and PNA lectin for detection. With this ELISA method, we could estimate the amounts of lubricin in OA SF to be in the range of 300–550 µg/ml (Fig. 4c). After incubation with CG (8–15 w/w%, enzyme to lubricin weight ratio), the amount of lubricin was decreased approximately 50% Fig. 3d).

### An endogenous 25-kDa lubricin fragment is found in SF from OA patients and is increased after CG incubation

**Figure 5:**
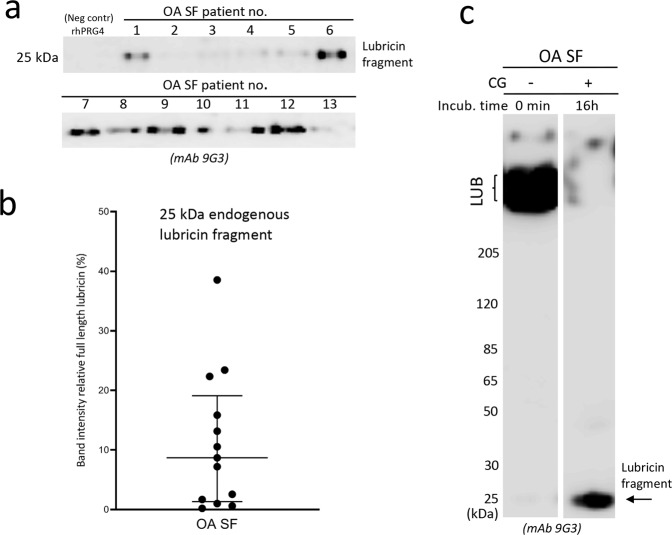
Identification of the 25 kDa glycosylated lubricin fragment from synovial fluid (SF) of OA patients. **(a)** Detection of an endogenous lubricin degradation fragment at 25 kDa in SF from OA patients. SF samples (2 μl) were analysed with SDS-PAGE, followed by western blot using mAb 9G3. Selected regions from two western blots showing examples of a lubricin degradation fragment at 25 kDa from 13 OA patients. The full-length western blots are displayed in Supplementary Fig. S5. **(b)** Semiquantification of the 25-kDa lubricin fragment in SF from 13 OA patients. The 25-kDa band intensities from western blot analyses are plotted relative to the full length lubricin band for every patient sample. rhPRG4 (1 μg) was used as negative control. **(c)** SF incubated with exogenous CG. SF (2 μL) from an OA patient was incubated with CG (44 ng) for 0 or 16 hours, followed by SDS-PAGE and detection with mAb 9G3. The original western blot together with analyses from two more patients are displayed in Supplementary Fig. S6.

We were interested to investigate if the 25 kDa mucin fragment generated from lubricin also could be found in SF, providing evidence that lubricin is degraded in its natural environment. Indeed, by increasing exposure time of the western blot using the mucin domain mAb 9G3, a single lubricin fragment at 25 kDa of endogenous origin was observed in SF from 13 OA patients without CG supplementation (Fig. 5a,b and Supplementary Fig. S5). The selected region from a western blot shows the lubricin degradation fragment at 25 kDa in SF from thirteen patients (Fig. 5a).

We could also show that the amount of the 25 kDa fragment was increased after addition of exogenous CG to SF (Fig. 5c and Supplementary Fig. S7). Degradation of full length lubricin was accompanied by a concomitant increase of a 25-kDa lubricin fragment, similar as was observed for rhPRG4 (Fig. 3a). We found that extending the incubation of lubricin with CG even for a longer period (>16 hours, results not shown) eventually decreased the intensity of the 25-kDa fragment. This indicated that CG was capable of digesting this component even further.

These experiments suggest that endogenous SF CG is at least partly responsible for generating the 25-kDa lubricin fragment found in OA patients.

### Proteomics and glycoproteomics analyses of rhPRG4 digested with CG

**Figure 6:**
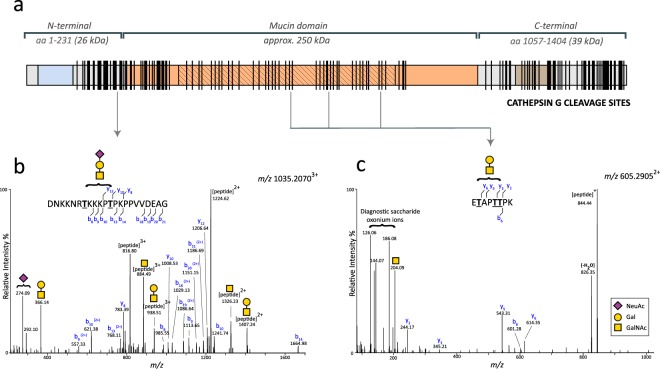
(**a**) Recombinant lubricin (rhPRG4) was digested with cathepsin G (CG), followed by analyses of the peptides with proteomics and glycoproteomics. Identified cleavage sites along the lubricin protein are represented by vertical lines. The complete peptide/glycopeptide lists are found in Supplementary Tables S1 and S2. **(b**,**c)** Higher-energy collisional dissociation (HCD) spectra of *O*-glycopeptides of rhPRG4 digested with CG, and analysed with LC/MS/MS. Diagnostic glycan oxonium ions are detected in the lower mass range (*m/z* 100–400).Fragment b/y-ions were detected without glycan substituents. Potential *O*-glycan sites (Ser/Thr) are underlined and in bold. **(b)** The glycopeptide at *m/z* 1035.2070 (3+) eluting at 14.3 min was deduced to have the sequence ‘DNKKNRTKKKPTPKPPVVDEAG’ and carry the Sialyl T-antigen (NeuAc-Gal-GalNAc). The glycopeptide originates from the lubricin *N-*terminal (aa positions 202–233). **(c)** The glycopeptide at *m/z* 605.2905(2+) eluting at 15.0–15.4 min was deduced to have the sequence ‘ETAPTTPK’ and carry the T-antigen (Gal-GalNAc). The peptide originates from the mucin domain and is found three times in lubricin (aa positions 615–622,703–710, 825–832).

In order to identify CG cleavage sites within lubricin, rhPRG4 was incubated with CG in PBS overnight at 37 °C at a ratio of 1:45 (enzyme to substrate, by weight), and the obtained degradation products analyzed with LC-MS/MS. We identified 135 non-glycosylated peptides and 72 glycopeptides in the size range of 6–37 amino acids, the majority from the *N*- and *C*-terminal of lubricin (Supplementary Tables S1 and S2). CG has been described to have a combination of tryptic and chymotryptic type specificity, but is also reported to cleave at other amino acids^40–42^. An overview of the cleavage sites in lubricin detected here are displayed in Fig. 6a. As a control experiment, we performed semi-tryptic searches of tryptic digests of rhPRG4, which revealed that 13 peptides could originate from other sources than CG digestion, for example autoproteolytic degradation. The most frequent CG cleavage site was C-terminal of lysine residues (33%), a site which has been reported previously for CG^40^. Many peptides were found to be overlapping within the same regions in the peptide backbone. CG cleavage sites within the glycosylated mucin domain were identified after manual evaluation of MS spectra.

The glycopeptides detected constituted glycoforms made up from 35 lubricin derived peptides, carrying glycans of different monosaccharide compositions, and consisting of Hex, HexNAc and NeuAc residues, matching simple core 1 type *O*-glycosylation that is produced in CHO cells from where the rhPRG4 was expressed (Supplementary Table S2). Higher-energy collisional dissociation (HCD) fragmentation did not reveal glycan site specific information, however all but one deduced peptide contained Thr or Ser residues. The remaining peptide D.MDYLPRVPN.Q from the C-terminal (aa 1122–1130) was detected as two glycoforms and proposed to be *O*-glycosylated on a Tyr residue (Supplementary Fig. S8). *N-*glycosylation was only found on the peptide L.RNGTVLAF.R (aa 1158–1165) detected as two glycoforms. The monosaccharide compositions revealed *N*-glycans of high mannose type (Supplementary Fig. S9). We have also detected this potential *N*-glycan site in tryptic digests of intact lubricin(data not shown). Mass spectra of three glycopeptides with the peptide sequences ‘ETAPTTPK’, ‘KEPAPTTPK’ and ‘KEPAPTTPKKPAPK’ from within the mucin repeat region are shown in Fig. 6c and Supplementary Figs S10 and S11, respectively. These repeat peptides were found 3, 17, and 2 times respectively in the mucin domain. Together with the identification of two nonglycosylated repeat peptides (‘KEPAPTTPKKPAPK’ and ‘SAPTTTKEPAPTTTK’, Supplementary Table S1), these data provided mass spectrometric evidence for that CG may cleave within the mucin region. The *N*-terminal derived glycopeptide with the sequence ‘K.DNKKNRTKKKPTPKPPVVDEAG.S’ (aa 202–223) contained two potential glycosylation sites (Thr208 and Thr213) and was detected as two glycoforms. The monosaccharide compositions of the first glycoform supported the presence of a T-antigen (Galβ1-3GalNAc) and the second glycoform supported the presence of the sialylated T-antigen (Fig. 6b). CG was also able to cleave this glycopeptide stretch further (Supplementary Table S2). The CG cleavage sites within amino acid sequence 202–223 are summarized by vertical lines: DN|K|K|NRTK|KKPTPKPPVV|D|E|A|G. This example illustrates that CG can cleave after many different amino acids in lubricin, however at low efficiency. This promiscuity in CG cleavage site specificity has been highlighted in studies with defined substrates^40^.

## Discussion

Despite being one of the most common and disabling joint diseases, OA pathogenesis and development are still largely unknown. The success of introducing recombinant lubricin for OA treatment in animal models^43,44^, indicates a role of lubricin in OA pathogenesis. To date, improper lubricin post-translational modifications, including glycosylation profile change^39^, forming complex with other matrix proteins^45^, and proteolytic cleavages are all reported in OA. This may ultimately alter the surface lubrication and contribute to disease development.

Here, we investigate the role of serine protease CG, with respect to its presence in OA SF as well as its degrading ability. CG has previously been suggested to be present in SF of OA-joints, using a colorimetric peptide substrate assay, specific for chymotryptic like proteases^37^. In this study, we validated the finding of CG in OA by detecting endogenous CG in SF from 16 patients using western blot (Fig. 2).

A complete degradation of rhPRG4, even for the putatively protected mucin domain, was observed after CG incubation, and this digestion can be enhanced by a prolonged time and increased enzyme levels within physiologically relevant concentrations (Fig. 3a,b and Fig. 4a). These data showed that CG is an efficient protease capable of degrading lubricin. Inspired by these results we monitored the effect of CG digestion of lubricin from SF from individual OA patients and from an SF pool from patients with RA. CG was found to readily degrade native lubricin, although at a slightly lower rate compared to rhPRG4 (Figs. 3c, 4a,b and 5c). The findings demonstrate the potent lubricin degrading ability of CG, both in a purified *in vitro* incubation system and in a more complex biological environment as in SF from OA patients. From our data we can speculate that three factors influence the CG degrading efficiency of lubricin:

1. Differences in glycosylation of the native lubricin variants (OA versus RA)^39^, and also for rhPRG4, the latter having CHO cell *O*-glycosylation.
2. CG degrading efficiency in SF is be affected by other proteins competing with lubricin as CG substrates.
3. SF serine protease inhibitors preventing CG activity.

The CG digests of both recombinant and native lubricin revealed a glycosylated lubricin degradation product of approximately 25 kDa, which was detected by western blot (using mAb 9G3 or PNA) and with the glycosensitive stain PAS staining (Figs. 3a, 4a,c, and 5c). The presence of glycosylation makes the fragment barely detectable with Coomassie protein stain. Tryptic digestion/MS analysis of this 25 kDa fragment to reveal its identity, did not generate any peptides or glycopeptides. The reason for this could be due to that CG was shown to display a promiscuous proteolytic nature, and that the monoclonal lubricin antibody 9G3, binds glycosylated forms of the sequence ‘KEPAPTTT’^12^, present multiple times in the mucin domain. Both these facts suggest that the 25 kDa fragment is not made up of a homogenous peptide stretch, making it hard to be identified. Inefficient tryptic digestion of dense and heterogeneous glycosylation, as well as MS ionization inefficiency of glycopeptides contributed to not being able of displaying the full spectrum of various lubricin glycopeptides constituting the 25 kDa fragment band.

With endogenous CG being present in SF of OA patients, we hypothesized that this protease could generate such a mucin domain containing fragment also *in vivo*. The detection of an endogenously produced 25 kDa lubricin fragment band with mAb 9G3 in SF from 13 late-stage OA patients (Fig. 5a,b) confirmed that lubricin was indeed degraded partially in OA SF by a CG like protease.

In all, our experiments thus supported that CG could be a protease candidate for proteolytic digestion of lubricin in SF. CG is a neutrophil serine protease, and neutrophils are the main immune cells found in SF in RA patients SF as an effect of RA associated inflammation. While lubricin degradation in RA was not specifically addressed here, our data suggests that lubricin degradation and neutrophil degradation may be highly relevant in RA pathology. Our observation that CG is present also in OA patients SF is in consistent with and extends previous findings that joint inflammation increases during OA and relates to that plasma neutrophil activation can serve as a biomarker of OA^46^. Our finding is in accordance with previous knowledge that CG is notably released during inflammatory processes and contributes to ECM degradation^35^. CG as a traditional immune activator and regulator, was proved to be responsible for pro-inflammatory IL-1 family cytokines activation in a recent research paper^47^. Cytokine IL-1β is found to be active during OA and induces inflammatory responses that cause protease activation and degradation of ECM proteins^28,48^. Furthermore, decreased amount of lubricin was reported by treating cartilage with IL-1β in animal models^21,49^. The present study proves a lubricin degradation ability of CG, which renders CG a potential role during OA, strengthens the importance of further understanding of CG in OA disease and provides the connection between inflammation and lubricin modifications during the OA pathologic process.

CG has been reported to be a promiscuous protease with both chymase and tryptase activity capable of cleaving both at charged amino acids (eg. Lys and Arg) as well as hydrophobic amino acids (eg Phe, Trp, and Leu)^40,42^. Proteomics and glycoproteomics of CG digestions of recombinant lubricin proved the enzyme to be an efficient protease, and gave an explanation for the degradation of lubricin observed in our western blots. We could detect peptides in the size range of 6–37 amino acids from both the *N*- and *C*-terminal, as well as the mucin domain, proving that CG digested lubricin at numerous sites in the protein backbone. 33% of the identified peptides contained *C*-terminal Lys residues (Supplementary Table S1 and S2). Hence, the Lys residues which are frequently found throughout the lubricin repeat region in the mucin domain must be obvious targets for digestion. Lys is the most common amino acid (165 aa of total of 1404) in lubricin after Thr and Pro, the two main constituents of the mucin domain. We detected three glycopeptide sequences originating from the mucin domain (Supplementary Table S2). Limited peptide coverage within the mucin domain is expected, since glycosylated peptides from the mucin domain are often more difficult to detect (high heterogeneity, poor ionization efficiency etc). These three glycopeptides occur multiple times in the mucin domain. Together with the detection of two nonglycosylated peptides from the same region (Supplementary Table S1), they provide ample evidence for that the mucin domain, which with glycosylation has an estimated size of 250 kDa (Fig. 1), can be degraded into 25-kDa and smaller fragments, as observed with western blot and proteomics.

In order to validate some of the lubricin peptides proposed to be formed by CG digestion in this study, we investigated the presence of ‘*in-vitro* formed’ autoproteolytic peptides in tryptic digests of recombinant lubricin (rhPRG4). Surprisingly, we did detect a small number of semitryptic peptides, which indicates that larger tryptic lubricin peptides may be degraded in the test tube to smaller semitryptic peptides that mistakenly can be assigned as a protease products. One example was the peptide H.VFMPEVTPDMDYLPR.V (aa1113–1127). The non-tryptic cleavage site at aa 1112 has previously been reported to be a cathepsin S cleavage site in tear fluid^21^, and equivalent site reported to be an endogenous protease cleavages in SF derived lubricin from horse^43^. Our data suggests an alternative explanation of post-proteolysis induced after tryptic digestion.

OA is a multifactorial disease, and beyond all doubt there is more than one factor that contributes to OA lubricin modifications. The present work demonstrates the potency of CG for lubricin degradation, providing the hypothesis that CG is involved and contributes to OA disease development. The involvement of CG as a neutrophil protease relevant in other arthritic diseases suggest that lubricin and CG degradation studies also would need to be expanded including other joint degrading diseases. The proteolytic cleavage product identified here has a potential to serve as local or systemic inflammatory biomarker for lubricin degradation.

## Materials and Methods

### Lubricin and synovial fluid samples

Synovial fluid (SF) samples were collected from 16 late-stage idiopathic OA patients (8 males and 8 females) subjected to knee replacement surgery. The mean age of the patients was 71 years (range 62–87 years). All individuals gave written consent and the procedure was approved by the ethics committee at Sahlgrenska University Hospital (ethical application 172–15). The SF samples were collected prior surgery, centrifuged, aliquoted and stored at −80 °C until assayed. Recombinant lubricin (rhPRG4, 1 mg/mL in Phosphate Buffered Saline (PBS) + 0.1% Tween 20) was obtained from Lubris BioPharma, USA. CG (22 ng/μL in PBS) was isolated from human leukocytes (Sigma-Aldrich, Germany). Purified native lubricin (SF Lub) was enriched from patients’ synovial fluid (pooled from RA patients, n = 10) by anion exchange chromatography and ethanol precipitation as described elsewhere^50^, and was quantified using lubricin sandwich ELISA as described below.

### *In vitro* digestion of lubricin by CG

rhPRG4 and lubricin purified from SF, 2–5 μg, were incubated with different concentrations (2.5–125 ng) of CG in at 37 °C in PBS (pH 7.4) for 30 minutes, 2 hours or over-night (16 hours). SF (2–2.5 μL) were incubated with CG (22–44 ng) in 37 °C for 1 or 2 hours, or a time series of 30 min and 16 hours (specific digestion conditions are indicated in figures). For positive control, CG activity was assessed using a fluorescence resonance energy transfer (FRET) substrate (Abz-EPFWEDQ-EDDnp)^51^. The assay was performed at 37 °C by mixing CG (10 nM, 25 µL) to the fluorescent substrate (20 µM, 25 µL). The reaction was buffered with 100 nM NaCl, 0.01 vol% Igepal CA-630 at pH 7.4^52^. Measurement of the increase in fluorescence was performed in regular intervals (Δt = 2 mins) and the plate was shaken regularly during the experiment. The results shown in Supplementary Fig S11.

### SDS-PAGE

Purified native lubricin and SF samples from CG incubations, and also SF samples (16 OA patient samples, 2 μl SF) used for screening of endogenous CG or lubricin degradation fragments, were reduced with 50 mM dithiothreitol (DTT) (Merck KGaA) followed by boiling at 95 °C for 15 minutes, and alkylation by 125 mM iodoacetamide (Sigma-Aldrich, St. Louis, MO, US) in dark for 45 minutes. Samples were analysed on NuPAGE Tris-acetate 3–8% gel (Invitrogen, Thermo Fisher Scientific, Waltham, MA, US). One μg of rhPRG4 or 22 ng CG (for endogenous CG assay) were included on each gel as controls. Molecular weight was compared to PageRuler Plus Prestained Protein Ladder (10 to 250 kDa, Thermo Fisher Scientific, USA). Gels were stained either with Coomassie brilliant blue R-250 (Bio-Rad Laboratories, Hercules, CA, US) or with Periodic Acid-Schiff (PAS) (Sigma-Aldrich) according to the manufacturers’ instructions. Lubricin band intensities were plotted and peak values were calculated by ImageJ (ImageJ 1.50i, USA)^53^.

### Western blot antibody and lectin staining

After electrophoresis, the gels were blotted to an Immobilon-P PVDF Membrane (Merck Millipore, Burlington, MA, US) using Trans-Blot SD Semi-Dry Transfer Cell (Bio-Rad Laboratories) at 200 mA for 80 minutes. After blocking with 1% bovine serum albumin (BSA) (VWR, Radnor, PA, US), the membranes were probed with 1 μg/mL mAb 9G3 against the glycosylated epitope ‘KEPAPTTT’ in the lubricin mucin domain (Merck KGaA, Darmstadt, Germany^12^) or polyclonal anti-CG antibody (Abcam, UK) 1/1000 diluted in assay buffer (1% BSA in PBS-Tween) for detection of endogenous CG in SF, followed by Horseradish Peroxidase (HRP) conjugated goat anti-mouse IgG (H + L) highly cross-adsorbed secondary antibody (Invitrogen, USA) 1/4000 diluted in assay buffer. For the lectin staining assay, blots were first probed by 1 μg/ml biotinylated Peanut Agglutinin (PNA, Vector laboratories, CA,USA) followed by 0.2 μg/ml HRP-streptavidin (Vector laboratories). After incubations, membranes were stained by WesternBright ECL Spray (Advansta, USA) and visualized in a luminescent image analyser (LAS-4000 mini, Fujifilm, Japan). Band intensities were calculated by ImageJ 1.50i^53^.

### Lubricin sandwich ELISA

An in-house ELISA method was set-up and validated for measuring lubricin concentrations in SF, adapted from others^54,55^. Monoclonal antibody 9G3 (1 μg/mL in PBS) was coated on 96-well Nunc-Immuno maxisorp plates (Thermo Fisher Scientific) at 4 °C over-night. After blocking with 3% BSA in PBS + 0.05% Tween, SF samples were added as a dilution series (1/50) in assay buffer (1% BSA in PBS-Tween) and incubated for 1 hour at room temperature (RT). Bound proteins were then incubated with biotinylated PNA (Vector laboratories) (1 μg/mL, 1 hour at RT), followed by HRP-streptavidin (Vector laboratories) at 0.1 μg/mL (1 hour at RT). Between each incubation, the wells were washed three times with PBS-Tween to remove unbound reagents. Proteins were stained with 1-Step Ultra TMB-ELISA Substrate Solution (Thermo Fisher Scientific) until blue colour was fully generated and the reaction stopped by adding 1 M H_2_SO_4_. Absorbances were read at 450 nm, and compared with a standard curve using recombinant lubricin (dilution series of rhPRG4 (1 mg/mL) in assay buffer). Samples were measured in duplicates and mean values are reported. The lubricin ELISA had an intra plate CV = 7.5% (n = 1 SF sample with 10 repeats) and a inter plate CV% = 7.8% (n = 1 SF sample, tested on 4 plates). Technical performance of the assay is summarized in Supplementary Table 3.

### LC-MS/MS and MS data analyses

rhPRG4 (10 μg in 10 μl PBS + 0.1% Tween 20) was incubated with CG (0.22 μg in 10 μL) in 37 °C under non-reducing conditions over night. Technical incubations experiments and MS analyses were performed in duplicates, with or without subsequent Tween removal (Pierce Detergent Removal Spin Column 125 μL, Thermo Fisher Scientific), where the latter approach was found be generating fewer detected peptides. The results reported (Supplementary Tables S1 and S2) are the combined result. For control experiments and semitryptic searches, rhPRG4 was digested with trypsin in-solution or in-gel as described elsewhere^56^. The peptides were desalted using C18 ziptips, followed by separation with LC-MS using in-house packed C18 columns at a flow rate of 200 nL/min, and a 45-min gradient of 5–40% buffer B (A: 0.1% formic acid, B:0.1% formic acid, 80% acetonitrile). The column was connected to an Easy-nLC 1000 system (Thermo Fisher Scientific, Odense, Denmark), a nano-electrospray ion source and a Q-Exactive Hybrid Quadrupole-Orbitrap Mass Spectrometer (Thermo Fisher Scientific). For full scan MS, the instrument was scanned *m/z* 350–2000, resolution 60000 (*m/z* 200), AGC target 3e6, max IT 20 ms, dynamic exclusion 10 sec. The twelve most intense peaks (charge states 2, 3, 4) were selected for fragmentation with higher-energy collisional dissociation (HCD). For MS/MS, resolution was set to 15000 (*m/z* 200), AGC target to 5e5, max IT 40 ms, and collision energy NCE = 27%.

Raw data files were searched against the human Uniprot protein database (downloaded 17.11.2017) using Peaks Studio 8.5 (Bioinformatics Solutions Inc., Waterloo, Canada). For peptide identification, mass precursor error tolerance was set to 5 ppm, and fragment mass error tolerance to 0.03 Da, enzyme: none; variable modifications: oxidation (M) and deamidation (NQ). Peptide-spectrum matches were filtered to 0.1% false discovery rate (Peaks peptide score > 25). Glycopeptide MS-MS spectra were selected with Peaks Studio software and/or manually using glycan diagnostic ions in the lower mass range (*m/z* 186, 204, 274, 292, 366). Spectra were evaluated manually, all major fragment ions were assigned and >4 b/y ions required in order to identify the peptide backbone. The assignments were aided by proteomics mining tools available free of charge (Findpept and PeptideMass (web.expasy.org); MS-product (Protein Prospector http://prospector.ucsf.edu). Mass precursor errors were less than 5 ppm.

### Statistics

All statistical analyses were performed using GraphPad Prism 8 for MacOS (GraphPad Software, San Diego, California USA, www.graphpad.com). Statistical difference was calculated by two-tailed Mann Whitney test or ordinary two-way ANOVA with Holm-Sidak’s multiple comparison.

### Ethical approval

All OA and RA patients gave informed consent and all the procedures were approved by the regional ethical review board in Gothenburg (172-15,13/5-2015). The study conformed to the ethical guidelines of the 1975 Declaration of Helsinki of research involving human subjects. All methods were performed in accordance with the relevant guidelines and regulations.

## Supplementary information

Supplementary information.

Supplementary information 2.

Supplementary information 3.

Supplementary information 4.
