# Author Correction: Cathepsin g Degrades Both Glycosylated and Unglycosylated Regions of Lubricin, a Synovial Mucin

**DOI:** 10.1038/s41598-020-77619-5

**Published:** 2021-02-15

**Authors:** Shan Huang, Kristina A. Thomsson, Chunsheng Jin, Sally Alweddi, André Struglics, Ola Rolfson, Lena I. Björkman, Sebastian Kalamajski, Tannin A. Schmidt, Gregory D. Jay, Roman Krawetz, Niclas G. Karlsson, Thomas Eisler

**Affiliations:** 1 Department of Medical Biochemistry and Cell Biology, Institute of Biomedicine, Sahlgrenska Academy, University of Gothenburg, Gothenburg, Sweden; 2 Science for Life Laboratory, Department of Oncology-Pathology, Clinical Proteomics Mass Spectrometry, Karolinska Institutet, Solna, Sweden; 3 Department of Clinical Sciences Lund, Orthopaedics, Faculty of Medicine, Lund University, Lund, Sweden; 4 Department of Orthopaedics, Institute of Clinical Sciences, The Sahlgrenska Academy, University of Gothenburg, Gothenburg, Sweden; 5 Department of Rheumatology and Inflammation Research, Institute of Medicine, Sahlgrenska Academy, University of Gothenburg, Gothenburg, Sweden; 6 Department of Molecular Skeletal Biology, Lund University, Lund, Sweden; 7 Biomedical Engineering Department, University of Connecticut Health Centre, Farmington, CT USA; 8 Department of Emergency Medicine, Warren Alpert Medical School and Division of Biomedical Engineering, School of Engineering, Brown University, Providence, RI USA; 9 Cell Biology and Anatomy, Cumming School of Medicine, University of Calgary, 3330 Hospital Drive NW, Calgary, AB T2N4N1 Canada; 10 McCaig Institute for Bone and Joint Health, University of Calgary, Calgary, AB T2N4N1 Canada; 11 Department of Clinical Sciences, Danderyd Hospital, Karolinska Institutet, Stockholm, Sweden

Correction to: *Scientific Reports* https://doi.org/10.1038/s41598-020-61161-5, published online 06 March 2020

The Acknowledgements section in this Article is incomplete.

“This study was funded by grants for the Swedish state under the agreement between the Swedish government and the county council, the ALF-agreement (ALFGBG-722391), the Swedish Research Council (621-2013-5895), Kung Gustav V:s 80-års foundation, Petrus and Augusta Hedlund’s foundation (M-2016-0353), AFA insurance research fund (dnr 150150) and IngaBritt and Arne Lundberg Foundation. Sofa Grindberg and Paula-Terese Kelly Pettersson at Danderyd’s Hospital and Lotta Falkendahl at University of Gothenburg are acknowledged for their assistance in collecting samples. Lubris BioPharma, LLC are acknowledged for providing rhPRG4. Prof Johan Bylund and Felix Klose at the Institute of Odontology, University of Gothenburg, are acknowledge for technical assistance in evaluating CG activity. Open access funding provided by University of Gothenburg.”

should read:

“This study was funded by grants for the Swedish state under the agreement between the Swedish government and the county council, the ALF-agreement (ALFGBG-722391), the Swedish Research Council (621-2013-5895), Kung Gustav V:s 80-års foundation, Petrus and Augusta Hedlund’s foundation (M-2016-0353), AFA insurance research fund (dnr 150150) and IngaBritt and Arne Lundberg Foundation. Sofa Grindberg and Paula-Terese Kelly Pettersson at Danderyd’s Hospital and Lotta Falkendahl at University of Gothenburg are acknowledged for their assistance in collecting samples. Lubris BioPharma, LLC are acknowledged for providing rhPRG4. Prof Johan Bylund and Felix Klose at the Institute of Odontology, University of Gothenburg, are acknowledge for technical assistance in evaluating CG activity. Prof Gregory D. Jay was supported by NIH 5P30GM122732. Open access funding provided by University of Gothenburg.”

