# Supplementary material for: Cathepsin g Degrades Both Glycosylated and Unglycosylated Regions of Lubricin, a Synovial Mucin
Source: Sci Rep. 2020 Mar 6;10:4215. doi: 10.1038/s41598-020-61161-5 (PMC7060204; doi:10.1038/s41598-020-61161-5)
Supplement: Supplementary file 1 — Supplementary information. [file 41598_2020_61161_MOESM1_ESM.pdf]

Supplementary Table S1

In-solution cathepsin G digest of recombinant lubricin (rhPRG4)

Peptides were analyzed with LC-MS/MS as described in Materials and Methods section

Data was searched against the Uniprot database and filtered to 0.1 % FDR with 'no enzyme specificity' settings using Peaks Studio 8.5 as described in Materials and Methods section.

Peptides which are nonspecific for the cathepsin G digest are labelled with an asterisk (*). These were also detected in semitryptic searches of tryptic digests of rhPRG4 (data not shown)

Peptide	Start	End	-10lgP	Mass	ppm	m/z	z	RT	Area	Scan	#Spec	PTM
K.KAPPPSGASQTIK.S	118	130	44.83	1280.709	1.5	427.9108	3	14.83	5.71E+08	5951	21	
K.STTKRSPKPPNKK.K	131	143	26.52	1467.852	0.5	367.9705	4	10.93	1.88E+07	3122	2	
K.RSPKPPNKKKTK.K	135	146	42.52	1407.867	0.9	352.9744	4	10.62	2.37E+08	2999	20	
K.RSPKPPNKKKTKK.V	135	147	41.99	1535.962	0	384.9979	4	11.06	3.40E+07	3206	4	
K.RSPKPPNKK.K	135	143	33.45	1050.63	0.5	351.2174	3	5.35	1.38E+08	1523	21	
K.TKKVIESEEITEEH.S	145	158	29.82	1670.836	0.7	557.9531	3	18.31	1.61E+07	8632	2	
K.KVIESEEITEEH.S	147	158	39.44	1441.694	0.8	721.8547	2	19.64	1.06E+09	9663	12	
K.KVIESEEIT.E	147	155	25.49	1046.55	0.1	524.2821	2	20.25	6.99E+07	10142	2	
K.VIESEEITEEH.S*	148	158	33.81	1313.599	1.1	657.8073	2	21.62	5.51E+08	11206	8	
H.SVSENQESSSSSSSSSSSTIRK.I*	159	181	27.61	2334.042	1.2	779.0222	3	14.61	5.02E+06	5794	1	
S.SSSSSSSSSSSTIRK.I	167	181	29.09	1473.691	0	492.2375	3	12.21	4.98E+06	3946	2	
S.SSSSSSSSSSTIRKIK.S	168	183	38	1627.838	-0.1	543.6198	3	14.81	2.64E+07	5939	2	
S.SSSSSSSSSSTIRK.I*	168	181	33.67	1386.659	0.1	463.2269	3	12.32	1.11E+07	4033	2	
S.SSSSSSSSSTIRKIK.S	169	183	36.56	1540.806	1.1	514.6097	3	14.65	4.21E+07	5820	1	
S.SSSSSSSSSTIRK.I*	169	181	31.58	1299.627	0.5	434.2163	3	12.21	2.79E+07	3942	3	
S.SSSSSSSSTIRKIK.S	170	183	33.8	1453.774	0.4	485.5987	3	14.48	5.25E+07	5689	4	
S.SSSSSSSSTIRK.I*	170	181	28.01	1212.595	0.2	405.2056	3	12.09	2.39E+07	3851	2	
S.SSSSSSSTIRKIK.S	171	183	31.53	1366.742	0.3	456.588	3	14.14	3.79E+07	5429	2	
S.SSSSSSSTIRK.I*	171	181	27.12	1125.563	0	376.1948	3	11.86	1.40E+07	3674	2	
S.SSSSSSTIRKIK.S	172	183	31.45	1279.71	-0.6	427.5769	3	14.03	3.55E+07	5344	3	
S.SSSSSTIRKIK.S	173	183	33.3	1192.678	-0.1	597.3459	2	13.59	3.48E+07	5015	2	
S.SSSSSTIRK.I*	173	181	26.29	951.4985	-3.3	476.755	2	11.13	4.17E+06	3244	2	
S.SSSSTIRKIK.S	174	183	29.17	1105.646	0	369.5558	3	13.34	2.62E+07	4819	2	
K.SSKNSAANRELQK.K	184	196	35.13	1431.743	-0.5	478.2547	3	12.46	5.74E+07	4141	2	
K.SSKN(+.98)SAANRELQK.K	184	196	30.48	1432.727	0.1	478.5829	3	13	7.22E+06	4555	1	Deamidatio
K.NSAANRELQK.K	187	196	27.22	1129.584	0.2	565.7994	2	13.22	2.98E+07	4728	4	
N.SAANRELQK.K	188	196	28.6	1015.541	0.5	508.778	2	13.06	1.02E+08	4597	2	
F.KVTTPDTSTTQHNK.V	232	245	46.16	1556.779	0.1	519.9338	3	13.14	2.72E+07	4660	3	
F.KVTTPDTSTTQHNKVSTSPK.I	232	251	45.7	2156.107	-0.2	719.7096	3	15	1.79E+07	6086	2	
F.KVTTPDTSTTQH.N	232	243	37.56	1314.642	0.6	439.2214	3	14.61	1.72E+07	5789	4	
F.KVTTPDTSTTQHN.K	232	244	35.78	1428.684	0.6	477.2357	3	14.5	4.96E+07	5708	2	
F.KVTTPDTSTTQHN(+.98)KVSTSPK.I	232	251	33.79	2157.091	-1.1	432.425	5	15.28	3.06E+06	6298	2	Deamidatio
F.KVTTPDTSTTQ.H	232	242	31.81	1177.583	0.4	589.7988	2	15.42	3.22E+07	6407	2	
K.VTTPDTSTTQHNKVSTSPK.I	233	251	43.55	2028.012	0.6	677.0118	3	15.67	2.44E+07	6598	1	
K.VTTPDTSTTQHNK.V	233	245	37.24	1428.684	0.7	477.2357	3	14.16	4.96E+07	5442	3	
K.VTTPDTSTTQH.N	233	243	32.39	1186.547	0.5	594.2809	2	15.76	2.54E+07	6663	2	
K.VTTPDTSTTQHN.K	233	244	31.34	1300.59	-1.4	651.3011	2	15.44	3.59E+07	6419	2	
S.KETSLTVN.K	272	279	25.18	890.4709	0.5	446.243	2	17.57	5.86E+06	8065	2	
L.TVNKETTVETK.E	277	287	38.78	1248.656	1	625.3359	2	13.57	8.06E+07	5000	4	
N.KETTVETK.E	280	287	25.1	934.4971	0.6	468.2561	2	11.27	7.22E+07	3322	3	
N.KQTSTDGKEKTTSAKETQ.S	294	311	46.92	1966.981	1	656.6682	3	11.22	1.60E+08	3295	2	
N.KQTSTDGKEKTTSAK.E	294	308	44.36	1608.832	0	403.2152	4	2.52	2.91E+07	719	1	
N.KQTSTDGKEKTTSA.K	294	307	42.14	1480.737	0.5	494.5865	3	11.01	1.42E+07	3175	1	
N.KQTSTDGKEKTTSAKETQ(+.98)SIEKTSAK.D	294	319	31.81	2812.43	3.8	469.7474	6	13.41	7.27E+06	4873	1	Deamidatio
S.TDGKEKTTSAKETQSIEK.T	298	315	46.52	1980.001	0.2	496.0076	4	13.37	2.30E+07	4844	2	
K.TTSAKETQSIEKT.S	304	316	43.51	1422.72	0.8	712.368	2	15.21	5.92E+07	6247	4	
K.TTSAKETQSIEK.T	304	315	41.92	1321.673	1.7	661.8447	2	14.33	2.23E+08	5575	8	
T.TSAKETQSIEK.T	305	315	31.26	1220.625	-0.1	407.8821	3	13.56	5.16E+06	4989	1	
T.SAKETQSIEKTSAK.D	306	319	37.68	1506.789	1.2	503.2709	3	12.95	5.86E+06	4513	2	
A.KETQSIEKTSAK.D	308	319	37.52	1348.72	-0.2	450.5804	3	12.31	6.21E+06	4023	2	
K.ETQSIEKTSAK.D	309	319	26.09	1220.625	-0.1	407.8821	3	13.13	1.10E+07	4652	2	
K.SAPTTPKEPAPTTTK.S	373	387	25.56	1525.799	-1.9	509.6059	3	16.38	4.70E+06	7139	2	
K.SAPTTTKEPAPTTTK.S	529	543	25.16	1529.794	-0.1	510.9384	3	14.88	2.16E+06	5993	1	
L.KEPAPTTPKKPAPK.E	718	731	46.35	1488.866	-0.3	373.2238	4	12.85	4.84E+07	4436	3	
H.KSPDESTPELS.A	871	881	26.37	1188.551	1	595.2834	2	19.1	3.82E+07	9243	2	
H.KSPDESTPEL.S	871	880	25.72	1101.519	1.4	551.7675	2	21.39	1.17E+08	11022	2	
K.SEDAGGAEGETPHM.L*	1094	1107	39.1	1386.536	1	694.2759	2	19.92	2.30E+07	9881	4	
H.MLLRPHVF.M	1107	1114	35.7	1011.569	0.2	506.7917	2	26.84	1.26E+09	15249	3	
H.M(+15.99)LLRPHVF.M	1107	1114	29.98	1027.564	0.2	514.7892	2	25.21	2.69E+08	14003	3	Oxidation (I
H.MLLRPHV.F	1107	1113	29.29	864.5004	-0.7	433.2572	2	22.45	5.33E+07	11857	2	
H.M(+15.99)LLRPHV.F	1107	1113	26.94	880.4953	0.9	441.2553	2	20.67	5.03E+06	10467	2	Oxidation (I
M.LLRPHVF.M	1108	1114	28.29	880.5283	-1.9	441.2706	2	24.33	4.34E+08	13318	7	
F.MPEVTPDM.D	1115	1122	30.68	918.3827	1	460.1991	2	26.42	4.13E+06	14930	2	
T.PDMDYLPRVPN.Q	1120	1130	38.88	1315.623	1.1	658.8195	2	28.14	4.59E+06	16213	2	
D.MDYLPRVPNQGI.I	1122	1133	46.68	1401.707	0	701.861	2	29.17	3.77E+07	16909	3	
D.MDYLPRVPN.Q	1122	1130	40.69	1103.543	-0.1	552.7789	2	28.1	2.16E+09	16181	10	
M.DYLPRVPN.Q	1123	1130	31.73	972.5029	0.3	487.2589	2	25.82	2.49E+08	14475	9	
N.GKPVDGLTTLR.N*	1148	1158	25.66	1155.661	0.7	578.8383	2	21.95	8.78E+06	11459	1	
W.MLSPFSPPSPARR.I	1172	1184	34.74	1441.75	1.2	721.8831	2	25.82	1.38E+08	14476	6	
W.MLSPFSPPSPAR.R*	1172	1183	30.28	1285.649	1.7	643.8328	2	28.02	2.43E+07	16120	3	
M.LSPFSPPSPARR.I	1173	1184	30.2	1310.71	3.1	656.3641	2	23.05	3.59E+08	12318	9	
M.LSPFSPPSPAR.R*	1173	1183	29.56	1154.608	-0.1	578.3114	2	25.12	8.46E+07	13932	4	
F.SPPSPARRITEVW.G	1177	1189	33.92	1494.794	0.3	748.4047	2	26.03	2.48E+09	14644	12	
F.SPPSPARR.I	1177	1184	25.78	866.4722	-0.2	434.2433	2	12.56	1.13E+08	4215	5	
E.VWGIPSPIDTVF.T	1188	1199	27.19	1329.697	1.4	665.8566	2	40.5	8.76E+06	25480	1	
V.WGIPSPIDTVF.T	1189	1199	26.24	1230.628	1.7	616.3225	2	39.57	5.27E+06	24826	1	
F.KDSQYWRF.T*	1212	1219	25.59	1128.535	-0.3	377.1855	3	26.57	1.65E+07	15042	1	
W.RFTNDIKDAGYPKPIFK.G	1218	1234	26.95	2009.073	-0.1	402.8219	5	24.22	6.87E+06	13227	1	
F.TNDIKDAGYPKPIFK.G	1220	1234	47.19	1705.904	4.6	569.6445	3	23.25	1.82E+09	12479	7	
F.TN(+.98)DIKDAGYPKPIFK.G	1220	1234	36.53	1706.888	3.2	569.9717	3	24.17	1.80E+07	13186	1	Deamidatio
F.TNDIKDAGYPKPI.F	1220	1232	33.51	1430.741	-0.8	477.9204	3	22.42	1.49E+07	11832	1	
F.TNDIKDAGYPKPIFKGF.G	1220	1236	30.59	1909.994	-2.5	637.6703	3	27.65	1.47E+07	15842	1	
T.NDIKDAGYPKPIFK.G	1221	1234	44.5	1604.856	0.8	535.9598	3	23.22	1.59E+08	12453	3	
T.NDIKDAGYPKPIF.K	1221	1233	35.73	1476.761	0.6	493.2613	3	27.18	7.83E+06	15487	1	

N.DIKDAGYPKPIFK.G	1222	1234	40.79	1490.813	0.3	746.4141	2	24.07	1.20E+08	13116	6	
N.DIKDAGYPKPIF.K	1222	1233	35.5	1362.718	0.9	682.367	2	28.15	1.12E+07	16224	2	
I.KDAGYPKPIFK.G	1224	1234	37.46	1262.702	-0.1	421.908	3	21.09	1.16E+07	10788	2	
K.DAGYPKPIFK.G	1225	1234	38.12	1134.607	2.8	568.3125	2	23.4	6.77E+09	12593	20	
K.DAGYPKPIFKGF.G	1225	1236	36.03	1338.697	2	670.3572	2	29.22	6.68E+06	16937	1	
K.DAGYPKPI.F	1225	1232	25.17	859.4439	-0.3	430.7291	2	22.17	3.24E+07	11631	2	
D.AGYPKPIFK.G	1226	1234	25.38	1019.58	-2.2	510.7963	2	23.42	3.55E+06	12610	2	
A.GYPKPIFK.G*	1227	1234	29.13	948.5432	-0.9	475.2784	2	23.48	2.39E+07	12657	3	
K.GFGGLTGQIVAAL.S	1235	1247	28.51	1202.666	1.1	602.3409	2	37.1	1.80E+08	22941	3	
K.GFGGLTGQIV.A	1235	1244	25.07	947.5076	-0.3	474.761	2	31.9	3.98E+07	18736	1	
Y.KNWPESVYF.F	1253	1261	26.08	1168.555	0.1	585.285	2	30.78	2.16E+08	17967	1	
Y.FFKRGGSIQQY.I	1261	1271	28.63	1329.683	-0.1	444.2348	3	21.5	3.94E+07	11113	2	
F.FKRGGSIQQY.I	1262	1271	27.29	1182.615	1.2	592.3152	2	18.5	3.13E+08	8786	6	
L.NYPVYGETTQVR.R	1289	1300	29.13	1425.689	-3.7	713.8491	2	23.46	9.15E+06	12640	2	
R.FERAIGPSQTH.T	1304	1314	28.71	1241.615	-1.5	414.8784	3	17.94	2.12E+07	8349	3	
F.ERAIGPSQTH.T	1305	1314	32.6	1094.547	0.7	365.8565	3	14.77	6.36E+05	5906	1	
R.AIGPSQTHTIR.I	1307	1317	39.4	1179.636	-1.9	394.2185	3	17.27	1.32E+08	7830	5	
R.AIGPSQTHTI.R	1307	1316	30.69	1023.535	1.2	512.7753	2	20.92	1.25E+07	10660	2	
R.AIGPSQTH.T	1307	1314	29.42	809.4031	-0.6	405.7086	2	15.64	3.22E+08	6575	8	
I.GPSQTHTIR.I	1309	1317	30.2	995.5148	1.4	498.7654	2	17.44	8.13E+05	7961	1	
T.HTIRIQYSPARL.A	1314	1325	26.91	1453.815	0.9	485.6128	3	23.33	2.01E+06	12541	1	
H.TIRIQYSPARL.A	1315	1325	36.27	1316.757	0.9	439.9265	3	25.55	8.91E+08	14268	7	
H.TIRIQYSPAR.L	1315	1324	36	1203.672	-1.4	402.2308	3	21.61	1.21E+07	11192	2	
H.TIRIQYSPARLA.Y	1315	1326	34.7	1387.794	-1.1	463.6046	3	25.23	1.57E+06	14017	1	
T.IRIQYSPARL.A	1316	1325	29.52	1215.709	0	406.2435	3	25.01	3.08E+06	13843	1	
I.RIQYSPARL.A	1317	1325	26.79	1102.625	-0.6	368.5486	3	22.11	3.67E+07	11589	2	
R.IQYSPARL.A	1318	1325	25.51	946.5236	-0.2	474.269	2	24.02	2.16E+08	13076	1	
L.AYQDKGVLH.N	1326	1334	31.51	1029.524	0.8	515.7698	2	17.19	1.03E+07	7766	2	
A.YQDKGVLH.N	1327	1334	26.49	958.4872	0.6	480.2512	2	16.72	2.97E+06	7399	1	
Y.QDKGVLH.N	1328	1334	25.24	795.4239	-0.2	398.7191	2	13.39	9.53E+07	4856	2	
K.VSILWRGLPNVVTSAISLPN(+.98)IRKPDGYDYYAFSKDQYY.N	1339	1376	32.53	4409.237	4.6	1103.322	4	35.85	4.26E+06	21950	1	Deamidatio
L.WRGLPN.V	1343	1348	30.55	741.3922	0.9	371.7037	2	23.38	1.50E+09	12578	9	
L.WRGLPNVVTS.A	1343	1352	27.63	1127.609	0.6	564.812	2	27.74	7.66E+08	15906	5	
W.RGLPNVVTSAI.S	1344	1354	34.26	1125.651	0	563.8326	2	28.39	3.77E+08	16383	4	
N.VVTSAISLPNIRKPDGY.D	1349	1365	28.26	1829.005	1	610.6761	3	27.9	3.05E+07	16027	1	
V.TSAISLPNIRKPDGY.D	1351	1365	40.62	1630.868	1.1	816.4421	2	26.63	1.24E+08	15091	4	
T.SAISLPNIRKPDGY.D	1352	1365	37.49	1529.82	1.3	510.948	3	26.7	3.13E+09	15144	12	
T.SAISLPNIRK.P	1352	1361	25.44	1097.656	-0.8	549.8347	2	24.01	2.74E+07	13064	3	
S.AISLPN(+.98)IRKPDGY.D	1353	1365	29.23	1443.772	0.2	482.2647	3	27.36	2.04E+07	15624	1	Deamidatio
S.AISLPNIRKPDGYDYY.A	1353	1368	28.3	1883.942	0.1	942.9781	2	29.01	8.35E+08	16809	3	
A.ISLPNIRKPDGY.D	1354	1365	32.61	1371.751	-0.3	458.2575	3	26.19	8.01E+07	14756	6	
I.SLPNIRKPDGYDYY.A	1355	1368	43.43	1699.821	1.9	850.9192	2	25.93	2.80E+08	14564	3	
L.PNIRKPDGY.D	1357	1365	38.04	1058.551	-0.3	530.2826	2	22.79	1.65E+07	12116	2	
N.IRKPDGY.D	1359	1365	26.16	847.4551	-0.2	424.7348	2	16.62	9.86E+07	7321	3	
Y.AFSKDQYY.N	1369	1376	31.31	1020.455	1.4	511.2356	2	22.37	1.59E+09	11793	6	
Y.YNIDVPSRTARAITTR.S	1376	1391	32.94	1832.986	2.2	459.2547	4	23.02	1.10E+08	12299	2	
Y.YNIDVPSRTAR.A	1376	1386	32.15	1290.668	0.3	646.3414	2	21.08	1.24E+08	10778	5	
Y.NIDVPSRTARAITTR.S	1377	1391	27.25	1669.922	1.6	418.4885	4	20.89	2.97E+09	10634	8	
Y.NIDVPSRTARAI.T	1377	1388	25.51	1311.726	-0.4	438.249	3	22.01	2.50E+08	11510	3	
N.IDVPSRTARAITTR.S	1378	1391	27.09	1555.879	0.5	389.9773	4	20.51	2.53E+08	10338	5	
R.SGQTLSK.V	1392	1398	25.09	719.3813	-0.5	360.6978	2	12.52	2.69E+07	4182	2	
