# Supplementary material for: Cathepsin g Degrades Both Glycosylated and Unglycosylated Regions of Lubricin, a Synovial Mucin
Source: Sci Rep. 2020 Mar 6;10:4215. doi: 10.1038/s41598-020-61161-5 (PMC7060204; doi:10.1038/s41598-020-61161-5)
Supplement: Supplementary file 2 — Supplementary information 2. [file 41598_2020_61161_MOESM2_ESM.pdf]

Supplementary Table S2

Glycopeptides detected from an 'in-solution' cathepsin G digest of recombinant lubricin (rhPRG4).

Peptides were analyzed with LC-MS/MS, and evaluated manually, as described in Materials and Methods section.

Peptides are listed with their glycan composition, and proposed amino acid attachment sites are underlined (Ser/Thr/Tyr/Asn).

Peptides that occur more than one time in the protein (repeated peptides) are labelled in bold.

Glycopeptides are listed with their adjacent amino acids separated by a dot ('.') . The abbreviation 'X' means that there are more than one possible amino acid option.

							GLYCAN COMPOSITION					CALCULATED NONGLYCOSYLATED PEPTIDE	
START	END	*m/z* (measured)	Charge	Ret time (min)	Scan no.	PEPTIDE	HexNAc	Hex	NeuAc	ΔDa	Δppm	[M+H]+	Repeated peptides(amino acid positions)
118	130	742.9028	2	15.19	6233;6370	K.KAPPPSGASQTIK.S	1	0	0	0.0028540	1.92	1281.7161	
118	130	495.6037	3	14.4;14.58	5625;5766;5897	K.KAPPPSGASQTIK.S	1	0	0	0.0010780	0.73	1281.7161	
118	130	823.9286	2	13.74	5128;5256;5531;6208	K.KAPPPSGASQTIK.S	1	1	0	0.0016540	1.00	1281.7161	
118	130	549.6213	3	14.45	5127;5666	K.KAPPPSGASQTIK.S	1	1	0	0.0010780	0.65	1281.7161	
118	130	646.6539	3	15.16	6211;6341	K.KAPPPSGASQTIK.S	1	1	1	0.0034780	1.79	1281.7161	
118	130	969.4764	2	15.17	6222;6352	K.KAPPPSGASQTIK.S	1	1	1	0.0018540	0.96	1281.7161	
202	219	547.1006	5	13.36	4833	K.DNKKNRTKKKPTPKPPVV.D	1	1	1	0.0015260	0.56	2075.2448	
202	220	479.4762	5	12.63	4269	K.DNKKNRTKKKPTPKPPVVD.E	1	0	0	0.0008260	0.35	2190.2717	
202	220	511.8882	5	12.52	4183	K.DNKKNRTKKKPTPKPPVVD.E	1	1	0	0.0080260	3.14	2190.2717	
202	220	712.3813	4	12.82;12.99;13.17	4270;4410;4544;4687	K.DNKKNRTKKKPTPKPPVVD.E	1	1	1	0.0041020	1.44	2190.2717	
202	220	570.1049	5	12.82;13.00;13.17	4411;4554;4688	K.DNKKNRTKKKPTPKPPVVD.E	1	1	1	-0.0038740	-1.36	2190.2717	
202	221	671.8676	4	12.80	4398	K.DNKKNRTKKKPTPKPPVVDE.A	1	1	0	0.0021020	0.78	2319.3143	
202	221	537.6949	5	12.65	4278	K.DNKKNRTKKKPTPKPPVVDE.A	1	1	0	-0.0010740	-0.40	2319.3143	
202	221	744.6417	4	13.36	4834	K.DNKKNRTKKKPTPKPPVVDE.A	1	1	1	0.0031020	1.04	2319.3143	
202	222	762.4017	4	14.37;14.55	5600;5742	K.DNKKNRTKKKPTPKPPVVDEA.G	1	1	1	0.0060020	1.97	2390.3514	
202	223	703.8828	4	13.06	4598	K.DNKKNRTKKKPTPKPPVVDEAG.S	1	1	0	0.0043020	1.53	2447.3729	
202	223	563.3073	5	13.02;13.20	4570;4713	K.DNKKNRTKKKPTPKPPVVDEAG.S	1	1	0	0.0023260	0.83	2447.3729	
202	223	1035.2064	3	14.29	5543	K.DNKKNRTKKKPTPKPPVVDEAG.S	1	1	1	0.0041780	1.35	2447.3729	
202	223	776.6573	4	14.45	5665;5938;6075	K.DNKKNRTKKKPTPKPPVVDEAG.S	1	1	1	0.0069020	2.22	2447.3729	
202	226	1120.9166	3	17.62	8102	K.DNKKNRTKKKPTPKPPVVDEAGSGL.D	1	1	1	-0.0027220	-0.81	2704.5104	
205	220	623.0892	4	12.86	4448	K.KNRTKKKPTPKPPVVD.E	1	1	1	0.0005020	0.20	1833.1069	
205	220	498.6725	5	12.90	4477	K.KNRTKKKPTPKPPVVD.E	1	1	1	-0.0010740	-0.43	1833.1069	
205	223	916.1514	3	14.38	5612	K.KNRTKKKPTPKPPVVDEAG.S	1	1	1	0.0040780	1.48	2090.2080	
205	223	687.3655	4	14.31;14.84	5559;5964	K.KNRTKKKPTPKPPVVDEAG.S	1	1	1	0.0046020	1.68	2090.2080	
206	220	623.3268	4	14.61	5788	K.NRTKKKPTPKPPVVDE.A	1	1	1	0.0033020	1.33	1834.0545	
206	223	873.4525	3	14.91	6016	K.NRTKKKPTPKPPVVDEAG.S	1	1	1	0.0022780	0.87	1962.1131	
206	223	655.3436	4	14.98	6072	K.NRTKKKPTPKPPVVDEAG.S	1	1	1	0.0119020	4.55	1962.1131	
206	223	524.4744	5	14.98	6074	K.NRTKKKPTPKPPVVDEAG.S	1	1	1	0.0022260	0.85	1962.1131	
208	223	835.4384	3	14.98	6076	N.RTKKKPTPKPPVVDEAG.S	1	1	1	0.0028780	1.15	1848.0702	
208	223	626.8303	4	14.98	6073	N.RTKKKPTPKPPVVDEAG.S	1	1	1	0.0016020	0.64	1848.0702	
210	220	470.2731	3	15.11	6179	K.KKPTPKPPVVD.E	1	0	0	0.0001780	0.13	1205.7252	
210	220	621.3227	3	15.97	6821	K.KKPTPKPPVVD.E	1	1	1	0.0007780	0.42	1205.7252	
210	221	664.3367	3	16.48	7217	K.KKPTPKPPVVDE.A	1	1	1	0.0001780	0.09	1334.7678	
210	223	707.0245	3	16.80	7464	K.KKPTPKPPVVDEAG.S	1	1	1	0.0049780	2.35	1462.8264	
252	267	791.4083	3	21.59	11178	K.ITTAKPINPRPSLPPN.S	1	1	1	0.0024780	1.04	1715.9803	
252	267	913.1203	3	21.26	10921	K.ITTAKPINPRPSLPPN.S	2	2	1	0.0063080	2.30	1715.9803	
252	272	964.1537	3	20.44	10287	K.ITTAKPINPRPSLPPNSDTSK.E	1	1	1	0.0050780	1.76	2234.2139	
252	272	723.3666	4	20.25	10141	K.ITTAKPINPRPSLPPNSDTSK.E	1	1	1	0.0031020	1.07	2234.2139	
252	272	814.6498	4	20.30	10181	K.ITTAKPINPRPSLPPNSDTSK.E	2	2	1	0.0037320	1.15	2234.2139	
316	326	495.2504	3	15.72;16.24	6638;6499	K.TSAKDLAPTSK.V	1	1	0	-0.0007220	-0.49	1118.6052	
316	326	592.2832	3	16.24	7831	K.TSAKDLAPTSK.V	1	1	1	0.0022780	1.28	1118.6052	
316	327	625.3057	3	18.60	8864	K.TSAKDLAPTSKV.L	1	1	1	0.0013780	0.74	1217.6736	
316	334	703.8680	4	18.53	8813	K.TSAKDLAPTSKVLAKPTPK.A	2	1	1	0.0053320	1.90	1953.1379	
316	334	817.1553	4	18.97	9145	K.TSAKDLAPTSKVLAKPTPK.A	2	2	2	0.0063320	1.94	1953.1379	
317	326	494.5906	3	16.77	7441	T.SAKDLAPTSKV.L	1	1	0	-0.0008220	-0.55	1116.63	
317	326	591.6233	3	17.64	8120	T.SAKDLAPTSKV.L	1	1	1	0.0018780	1.06	1116.63	
317	334	719.1201	4	17.60	8090	T.SAKDLAPTSKVLAKPTPK.A	2	2	1	0.0086320	3.00	1852.09	
320	326	694.3148	2	15.55	6508;6562	K.DLAPTSK.V	1	1	1	0.0013540	0.98	731.39	
320	334	493.7768	4	18.51	8788	K.DLAPTSKVLAKPTPK.A	2	0	0	0.0005320	0.27	1565.9261	
320	334	863.1029	3	18.67	8916	K.DLAPTSKVLAKPTPK.A	2	2	1	0.0083080	3.21	1565.9261	
320	334	647.5783	4	18.71	8943	K.DLAPTSKVLAKPTPK.A	2	2	1	0.0055320	2.14	1565.9261	
320	334	607.0639	4	19.20	9321	K.DLAPTSKVLAKPTPK.A	2	1	1	0.0007320	0.30	1565.9261	
326	333	406.8990	3	13.70	5099;6852	K.VLAKPTPK.A	1	1	0	-0.0003220	-0.26	853.5506	
326	333	503.9305	3	16.00	6848	K.VLAKPTPK.A	1	1	1	-0.0012220	-0.81	853.5506	
326	333	755.3931	2	16.01	6852	K.VLAKPTPK.A	1	1	1	0.0007540	0.50	853.5506	
327	333	470.9079	3	15.31	6322	V.LAKPTPK.A	1	1	1	-0.0006220	-0.44	754.4822	
402	410	542.2612	3	15.11	6174	**X.KEPAPTTPK.X**	1	1	1	0.0003780	0.23	968.5411	402-10;433-41;449-57;472-80;496-504;558-66;566-74;582-90;606-14;678-86;686-94;694-702;718-26;762-71;771-79;787-95;832-40
402	410	812.8888	2	15.11	6178	**X.KEPAPTTPK.X**	1	1	1	0.0016540	1.02	968.5411	
615	622	524.2638	2	15.71	6627	**K.ETAPTTPK.X**	1	0	0	-0.0001460	-0.14	844.4411	615-622;703-710;825-832
615	622	605.2905	2	15.19	6236	**K.ETAPTTPK.X**	1	1	0	0.0004540	0.38	844.4411	615-622;703-710;825-832
615	622	750.8367	2	16.43	7174	**K.ETAPTTPK.X**	1	1	1	-0.0025460	-1.70	844.4411	615-622;703-710;825-832
718	731	423.9937	4	13.37;13.54;13.71;14.07	4974;5373;5109;4839	**L.KEPAPTTPKKPAPK.E**	1	0	0	-0.0000980	-0.06	1489.8737	718-731;787-800
718	731	564.9890	3	13.75;14.56;14.74	5140;5755;5888	**L.KEPAPTTPKKPAPK.E**	1	0	0	-0.0006220	-0.37	1489.8737	718-731;787-800
718	731	464.5075	4	12.99;13.17;14.64	4545;4686;5843	**L.KEPAPTTPKKPAPK.E**	1	1	0	0.0023020	1.24	1489.8737	718-731;787-800
718	731	619.0070	3	13.02;13.20	4573;4714	**L.KEPAPTTPKKPAPK.E**	1	1	0	-0.0025460	0.31	1489.8737	718-731;787-800
718	731	1073.5551	2	14.68	5840	**L.KEPAPTTPKKPAPK.E**	1	1	1	0.0016540	0.77	1489.8737	718-731;787-800
718	731	716.0397	3	14.62;14.79	5799;5925	**L.KEPAPTTPKKPAPK.E**	1	1	1	0.0032780	1.53	1489.8737	718-731;787-800
718	731	537.2816	4	14.41;14.59;14.78	5638;5778;5912	**L.KEPAPTTPKKPAPK.E**	1	1	1	0.0033020	1.54	1489.8737	718-731;787-800
718	731	430.0257	5	14.59;14.78	5779;5913	**L.KEPAPTTPKKPAPK.E**	1	1	1	-0.0018740	-0.87	1489.8737	718-731;787-800
718	731	474.7636	4	13.44;13.80;13.88	4898;5177;5229	**L.KEPAPTTPKKPAPK.E**	2	0	0	0.0001320	0.07	1489.8737	718-731;787-800
718	731	515.2770	4	13.62;13.97	5039;5297	**L.KEPAPTTPKKPAPK.E**	2	1	0	0.0009320	0.45	1489.8737	718-731;787-800
718	731	588.0514	4	14.83	5952	**L.KEPAPTTPKKPAPK.E**	2	1	1	0.0031320	1.33	1489.8737	718-731;787-800
718	731	628.5643	4	14.26;14.62	5523;5801	**L.KEPAPTTPKKPAPK.E**	2	2	1	0.0019320	0.77	1489.8737	718-731;787-800
718	731	837.7499	3	14.36	5799	**L.KEPAPTTPKKPAPK.E**	2	2	1	0.0017080	0.68	1489.8737	718-731;787-800
718	731	934.7829	3	14.78;14.96	5915;6055	**L.KEPAPTTPKKPAPK.E**	2	2	2	0.0053080	1.89	1489.8737	718-731;787-800
718	731	701.3372	4	14.71;14.87;15.04;15.22	5851;5990;6124;6262	**L.KEPAPTTPKKPAPK.E**	2	2	2	-0.0018680	-0.67	1489.8737	718-731;787-800
718	731	561.2717	5	14.89	6004	**L.KEPAPTTPKKPAPK.E**	2	2	2	0.0005560	0.20	1489.8737	718-731;787-800
871	887	1089.5138	2	19.57	9611	H.KSPDESTPELSAEPTPK.A	1	1	0	0.0019540	0.90	1812.8862	
871	887	1235.0596	2	20.34	10209	H.KSPDESTPELSAEPTPK.A	1	1	1	-0.0018460	-0.75	1812.8862	
871	887	823.7111	3	20.24	10128	H.KSPDESTPELSAEPTPK.A	1	1	1	0.0049780	2.02	1812.8862	
880	887	749.8493	2	17.19	7763	E.LSAEPTPK.A	1	1	1	0.0019540	1.30	842.4618	
882	887	649.7911	2	13.50	4947	S.AEPTPK.A	1	1	1	0.0016540	1.27	642.3457	
1073	1087	932.1101	3	16.19	6992	M.LQTTTRPNQTPNSKL.V	3	3	0	0.0059380	2.13	1698.9133	
1073	1087	1029.1414	3	17.12	7711	M.LQTTTRPNQTPNSKL.V	3	3	1	0.0044380	1.44	1698.9133	
1073	1087	1126.1714	3	17.72;17.90;18.07	8179;8315;8449	M.LQTTTRPNQTPNSKL.V	3	3	2	-0.0009620	-0.28	1698.9133	
1073	1087	1223.2062	3	18.54	8812	M.LQTTTRPNQTPNSKL.V	3	3	3	0.0080380	2.19	1698.9133	
1073	1087	917.6573	4	18.57	8838	M.LQTTTRPNQTPNSKL.V	3	3	3	0.0113620	3.10	1698.9133	
1073	1087	837.7497	3	16.77	7438	M.LQTTTRPNQTPNSKL.V	4	0	0	0.0037680	1.50	1698.9133	
1073	1087	891.7679	3	16.69	7373	M.LQTTTRPNQTPNSKL.V	4	1	0	0.0055680	2.08	1698.9133	
1073	1087	1042.8168	3	17.42	7945	M.LQTTTRPNQTPNSKL.V	4	2	1	0.0040680	1.30	1698.9133	
1073	1087	1139.8500	3	18.32	8643	M.LQTTTRPNQTPNSKL.V	4	2	2	0.0082680	2.42	1698.9133	
1073	1087	1096.8350	3	16.90	7542	M.LQTTTRPNQTPNSKL.V	4	3	1	0.0058680	1.78	1698.9133	
1073	1087	895.6526	4	17.74	8192	M.LQTTTRPNQTPNSKL.V	4	3	2	0.0085920	2.40	1698.9133	
1073	1087	1290.8986	3	18.40	8708	M.LQTTTRPNQTPNSKL.V	4	3	3	0.0058680	1.52	1698.9133	
1073	1087	968.4262	4	18.39	8696	M.LQTTTRPNQTPNSKL.V	4	3	3	0.0075920	1.96	1698.9133	
1122	1130	880.8947	2	25.61	14311	D.MDYLPRVPN.Q	1	1	1	0.0039540	2.25	1104.5506	
1122	1130	684.6301	3	25.69	14372	D.MDYLPRVPN.Q	1	1	2	0.0021780	1.06	1104.5506	
1122	1130	1026.4418	2	25.72	14397	D.MDYLPRVPN.Q	1	1	2	0.0027540	1.34	1104.5506	
1158	1165	1047.4613	2	24.38	13357	L.RNGTVLAF.R	2	5	0	0.0025840	1.23	877.4900	
1158	1165	1128.4889	2	24.20	13213	L.RNGTVLAF.R	2	6	0	0.0049840	2.21	877.4900	
