# Supplementary material for: Cathepsin g Degrades Both Glycosylated and Unglycosylated Regions of Lubricin, a Synovial Mucin
Source: Sci Rep. 2020 Mar 6;10:4215. doi: 10.1038/s41598-020-61161-5 (PMC7060204; doi:10.1038/s41598-020-61161-5)
Supplement: Supplementary file 3 — Supplementary information 3. [file 41598_2020_61161_MOESM3_ESM.pdf]

Supplementary Table S3. ELISA technical performance

Technical validation test	Result
Intra-assay variation, CV%	7.5
Inter-assay variation, CV%	7.8
Dilution linearity of recombinant lubricin, recovery rate	1:30 to 1:480, 112%
Dilution linearity of synovial fluid, recovery rate	1:20 to 1:160, 134%
Spike recovery in human synovial fluid	136% (high), 73% (medium), 85% (low)
Spike recovery in PBS (diluent)	116% (high), 80% (medium), 92% (low)
Slope of standard curve, CV%	7.4
