# Supplementary material for: Cathepsin g Degrades Both Glycosylated and Unglycosylated Regions of Lubricin, a Synovial Mucin
Source: Sci Rep. 2020 Mar 6;10:4215. doi: 10.1038/s41598-020-61161-5 (PMC7060204; doi:10.1038/s41598-020-61161-5)
Supplement: Supplementary file 4 — Supplementary information 4. [file 41598_2020_61161_MOESM4_ESM.pdf]

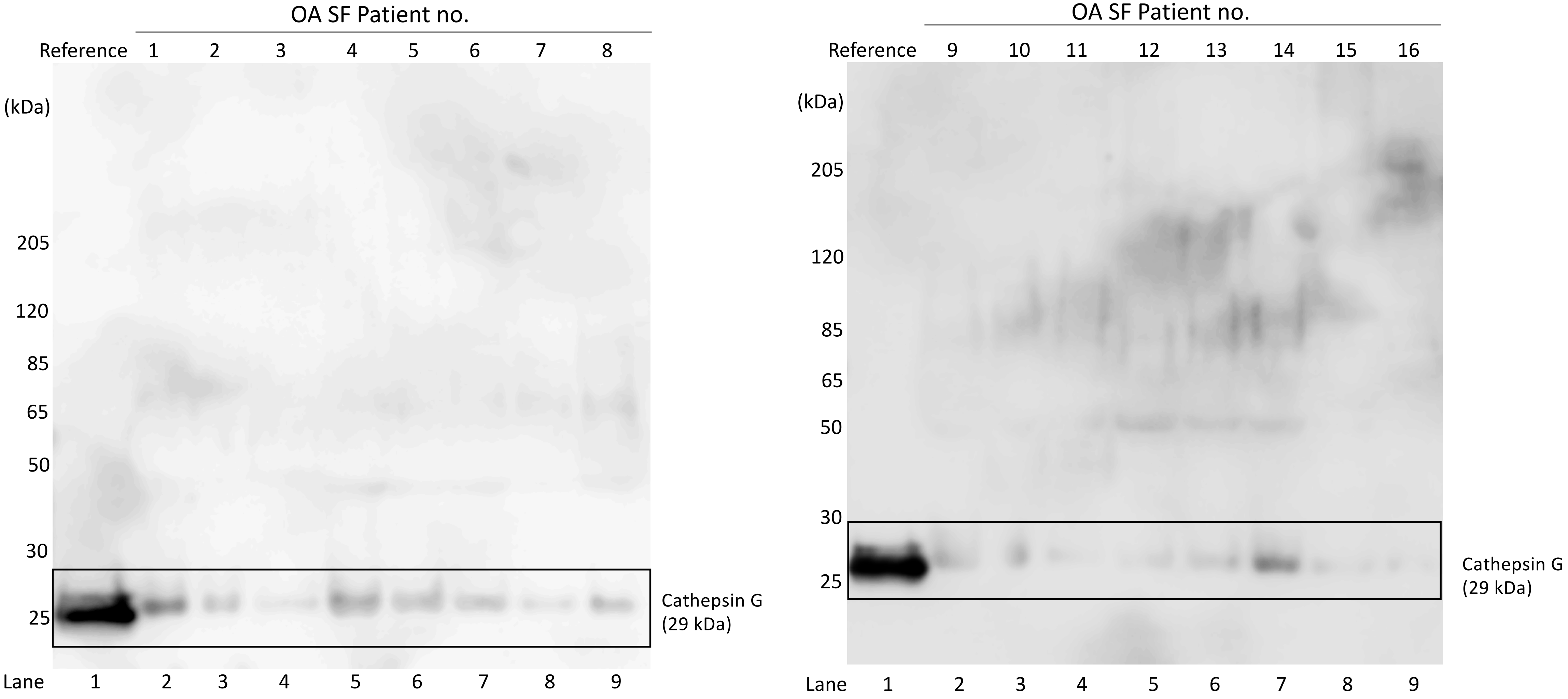

Supplementary Fig S1. **Endogenous Cathepsin G (CG) in synovial fluid (SF) from 16 OA patients**. SF was analysed with SDS-PAGE followed by western blot using a polyclonal anti-CG antibody (see Materials and Methods section). The reference compound was 22 ng CG. The selection of the blots displayed in Figure 2 are marked with a box.

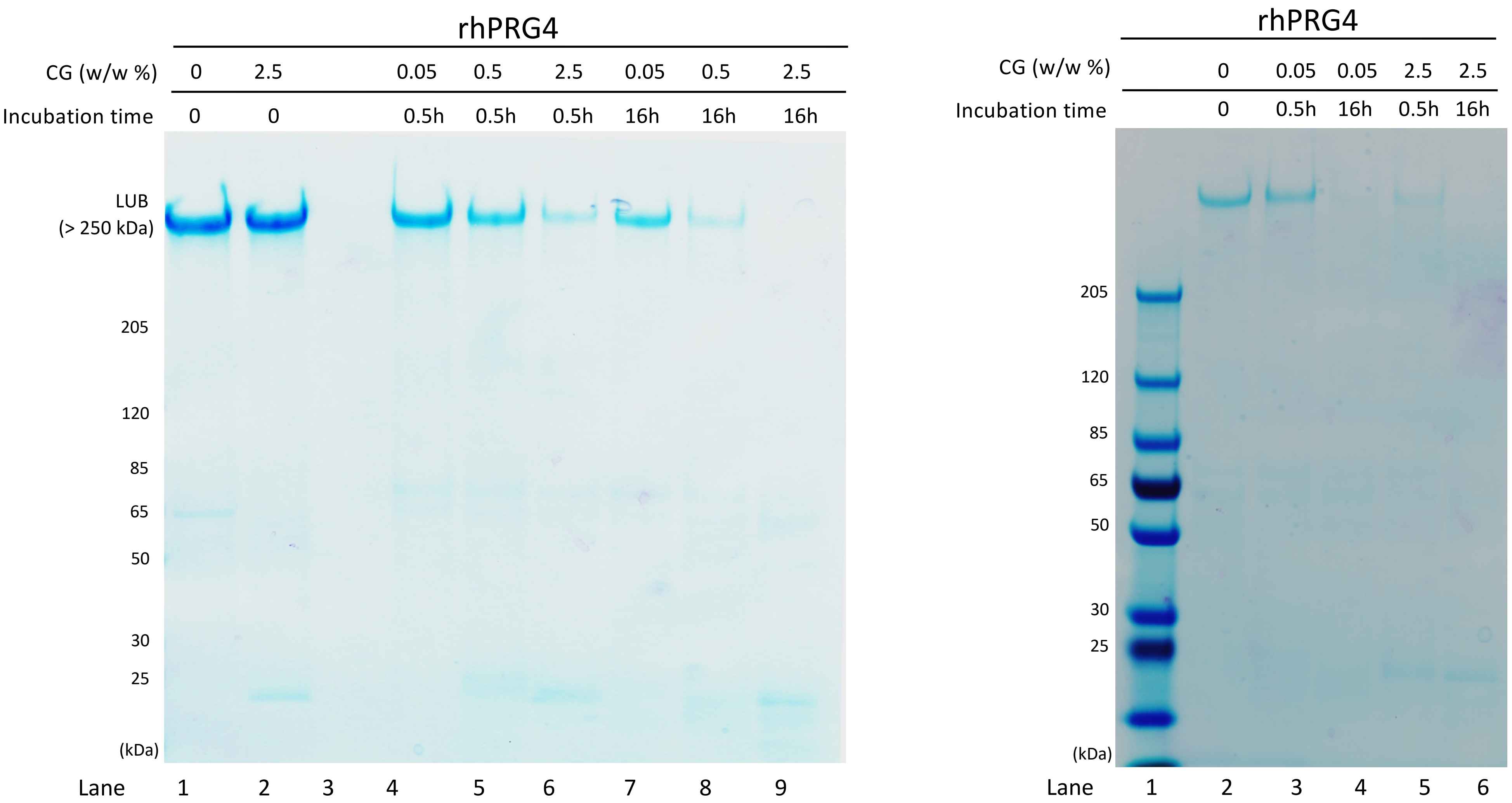

Supplementary Figure S2. Cathepsin G degradation of recombinant lubricin. Recombinant lubricin (rhPRG4) (2.5 μg) was incubated with cathepsin G (CG) in PBS at 37°C for 0, 0.5 or 16 hours with different CG/rhPRG4 weight to weight ratios. After reduction and alkylation, the samples were separated on tris-acetate gels (3-8%) and stained with Coomassie Blue (as described in Materials and Methods section).

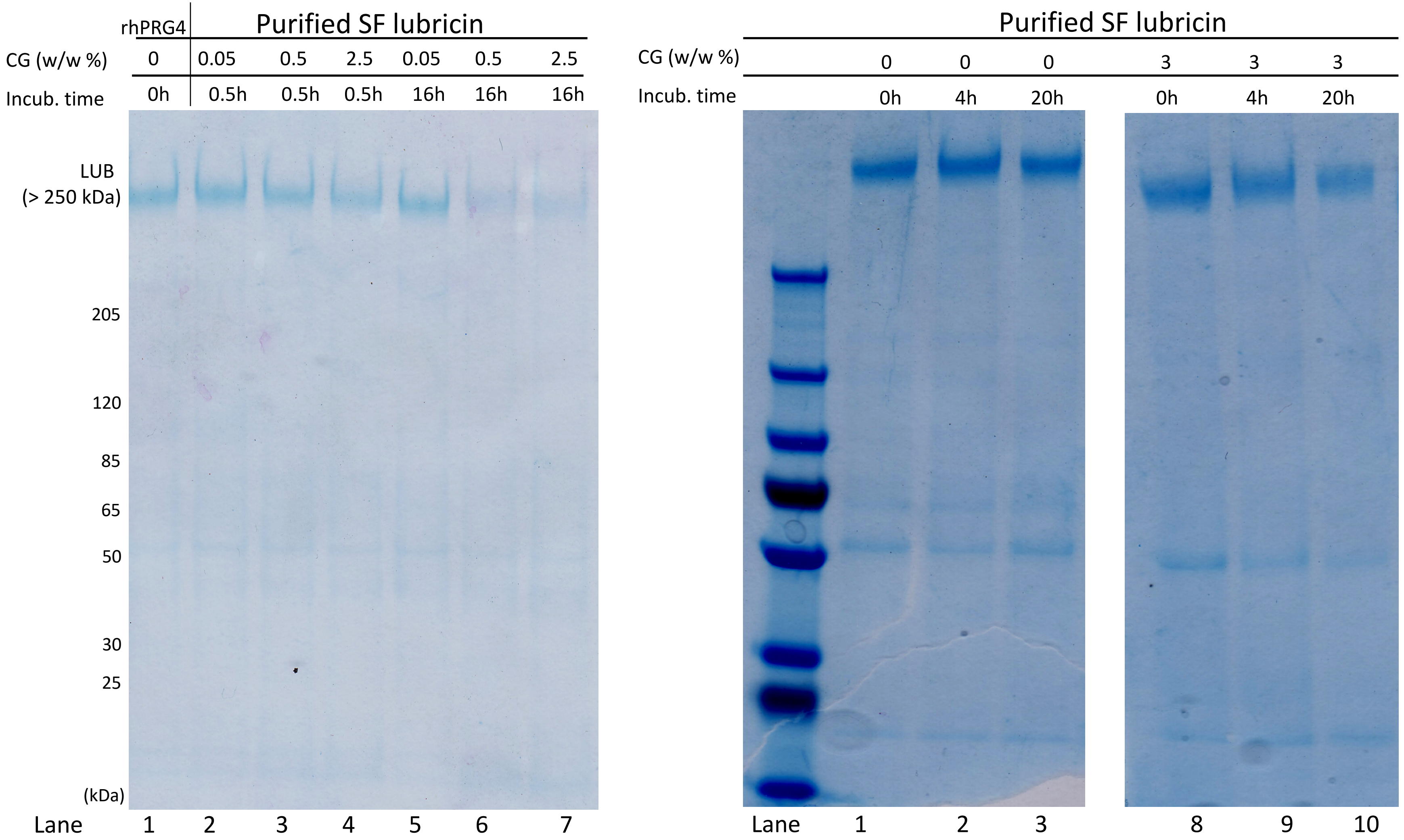

Supplementary Figure S3. Cathepsin G degradation of native lubricin. Lubricin (2.5 μg) purified from synovial fluid was incubated with cathepsin G (CG) in PBS at 37°C for incubation times of 0-20 hours and with different CG/lubricin weight to weight ratios. After reduction and alkylation, the samples were separated on tris-acetate gels (3-8%) and stained with Coomassie Blue (as described in Materials and Methods section).

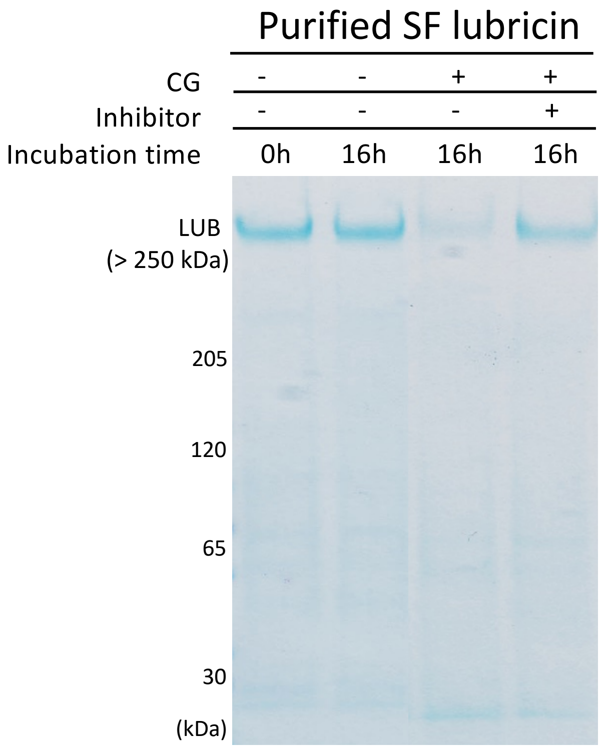

Supplementary Figure S4. The degradation of lubricin was abolished by the presence of cathepsin G inhibitor. Purified native lubricin (2.5 μg) from synovial fluid (SF) was incubated with or without cathepsin G (2.5 w/w%) and with or without cathepsin G inhibitor (1 μg, Abcam, UK) in PBS at 37°C over-night for 16 hours. After reduction and alkylation, the samples were separated on tris-acetate gels (3-8%), and stained with Coomassie Blue (as described in Materials and Methods section).

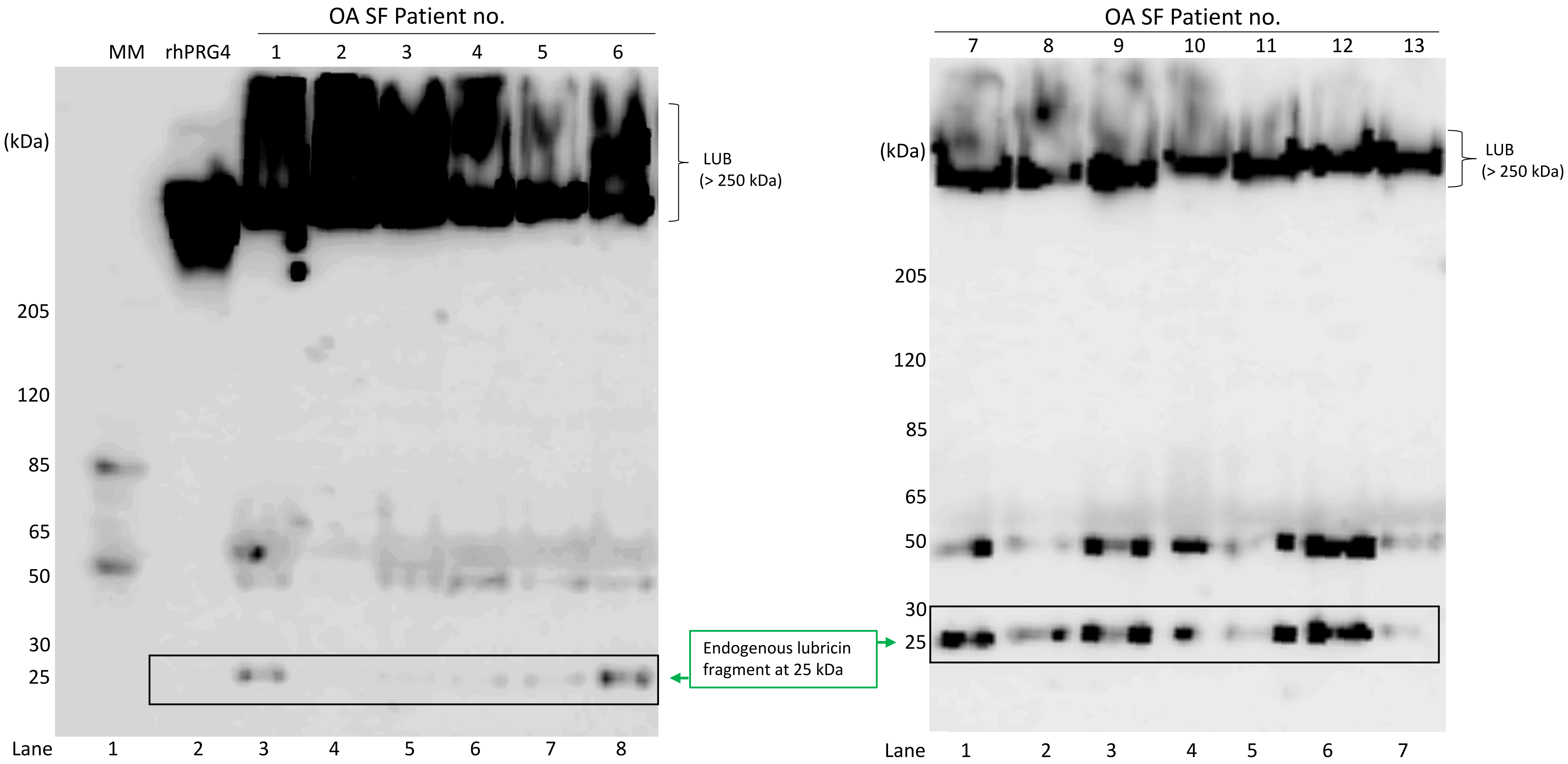

Supplementary Fig S5. **Identification of the endogenously formed 25 kDa glycosylated lubricin fragment from synovial fluid (SF) of 13 OA patients.** SF samples (2 µl) were analysed with SDS-PAGE, followed by western blot using mAb 9G3 (as described in Materials and Methods section). rhPRG4 (1 µg) was used as negative control. MM= molecular marker. The selection of the blots displayed in Figure 5a are marked with boxes.

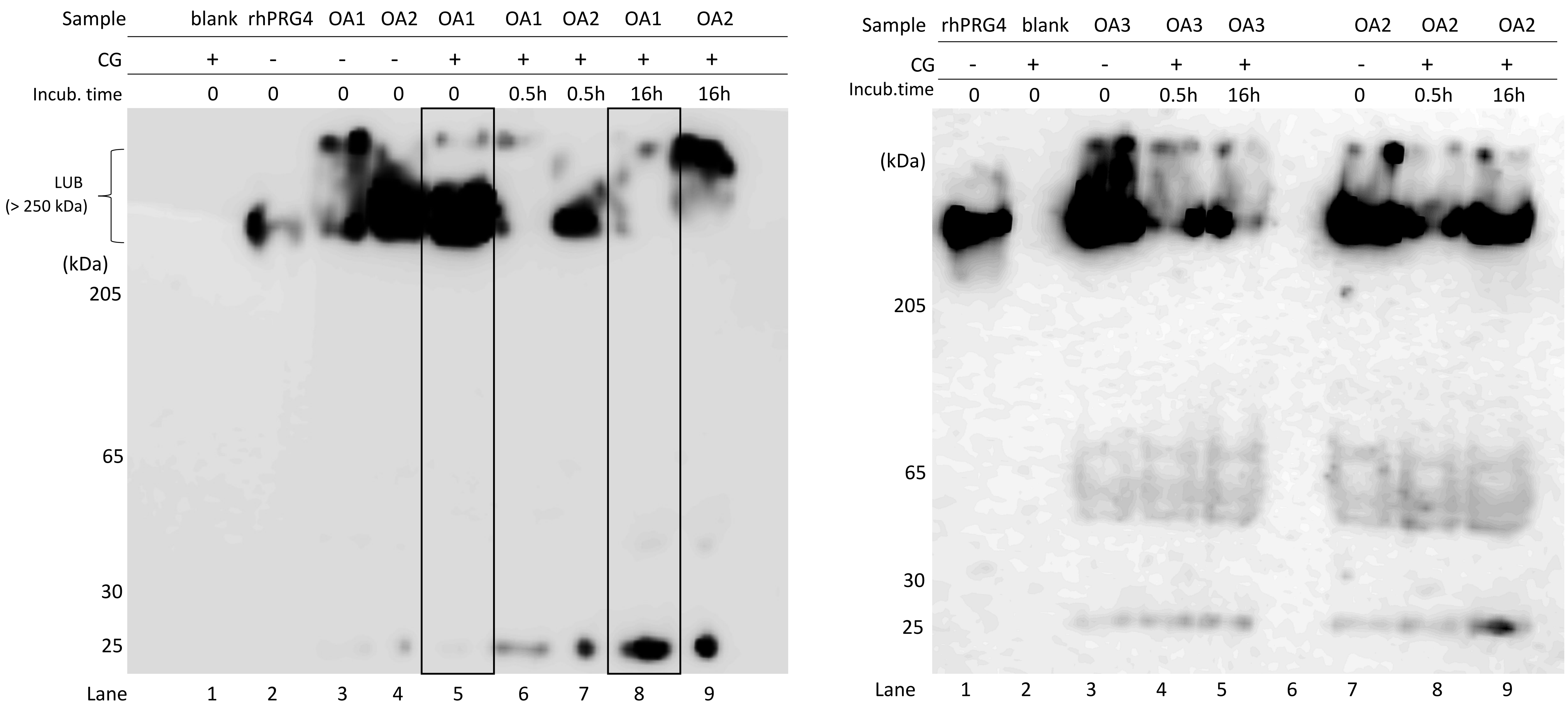

Supplementary Figure S6. Degradation of lubricin in synovial fluids from OA patients by exogenous supply of cathepsin G (CG). SF (2 µL) from three OA patients (OA1, OA2 and OA3) were incubated with CG (44 ng) for 0, 0.5 or 16 hours. After reduction and alkylation, the samples were separated on tris-acetate gels (3-8%), and developed with western blot using mAb 9G3 (as described in Materials and Methods section). The selections of the blot displayed in Figure 5c are marked with boxes.

D.MDYLPRVPN.Q + HexNAc-Hex-NeuAc
Amino acid position in sequence: 1122-1130
Obs. *m/z* 880.8947 (2+)
Calc. $[M+H]^+$ = 1760.7782 (Δppm= 2.25)

Supplementary Figure S7
HCD spectra of a *O*-glycopeptide (proposed Tyr O-glycosylation) from the mucin domain of recombinant lubricin (rhPRG4) digested with cathepsin G, and analysed with LC/MS/MS using a Qexactive instrument. Analytical conditions are described in Materials and Methods. Diagnostic glycan ions are detected in the lower mass range (*m/z* 100-400). b/y-ions are detected without glycan substituents.

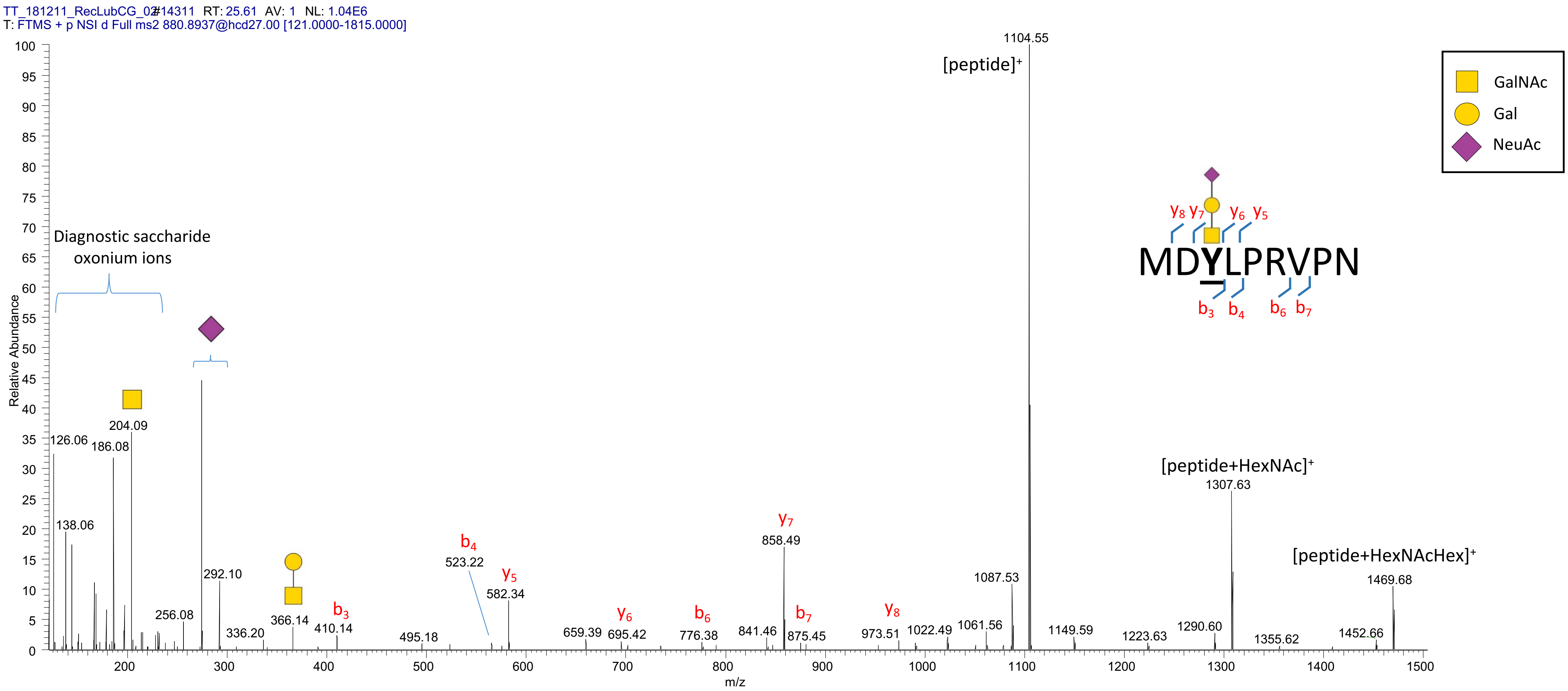

L.RNGTVLAF.R+ HexNAc(2) Hex(5)
Amino acid position in sequence: 1158-1165
Obs. *m/z* 1047.4613 (2+)
Calc. $[M+H]^+$ = 2093.9127 (Δppm= 1.23)

Supplementary Figure S8
HCD spectra of a *N*-glycopeptide from the mucin domain of recombinant lubricin (rhPRG4) digested with cathepsin G, and analysed with LC/MS/MS using a Qexactive instrument. Analytical conditions are described in Materials and Methods. b/y-ions are detected with or without glycan substituents as annotated.

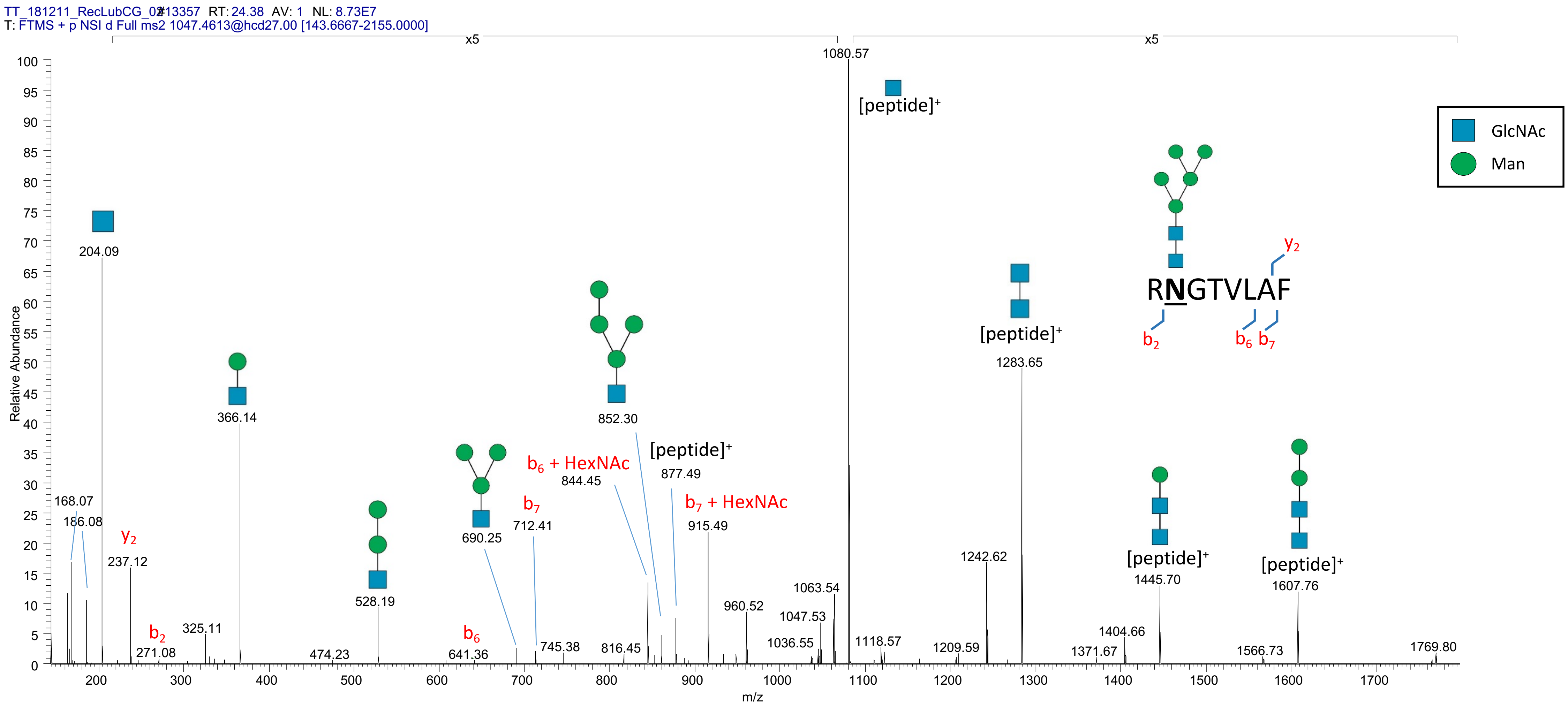

KEPAP**T****T**PK+ HexNAc-Hex-NeuAc

Amino acid positions in sequence: 402-10;433-41;449-57;472-80;496-504;558-66;566-74;582-90;606-14;678-86;686-94;694-702;718-26;762-71;771-79;787-95;832-40

Observed *m/z:* 542.2612 (3+)

Calc. $[M+H]^+$ = 1624.7687 (Δppm= 0.23)

Supplementary Figure S9

HCD spectra of an *O*-glycopeptide from the mucin domain of recombinant lubricin (rhPRG4) digested with cathepsin G, and analysed with LC/MS/MS using a Qexactive instrument. Analytical conditions are described in Materials and Methods. Diagnostic glycan ions are detected in the lower mass range (*m/z* 100-400). b/y-ions are detected without glycan substituents if not annotated elsewise. Potential *O*-glycan sites (Ser/Thr) are underlined and in bold.

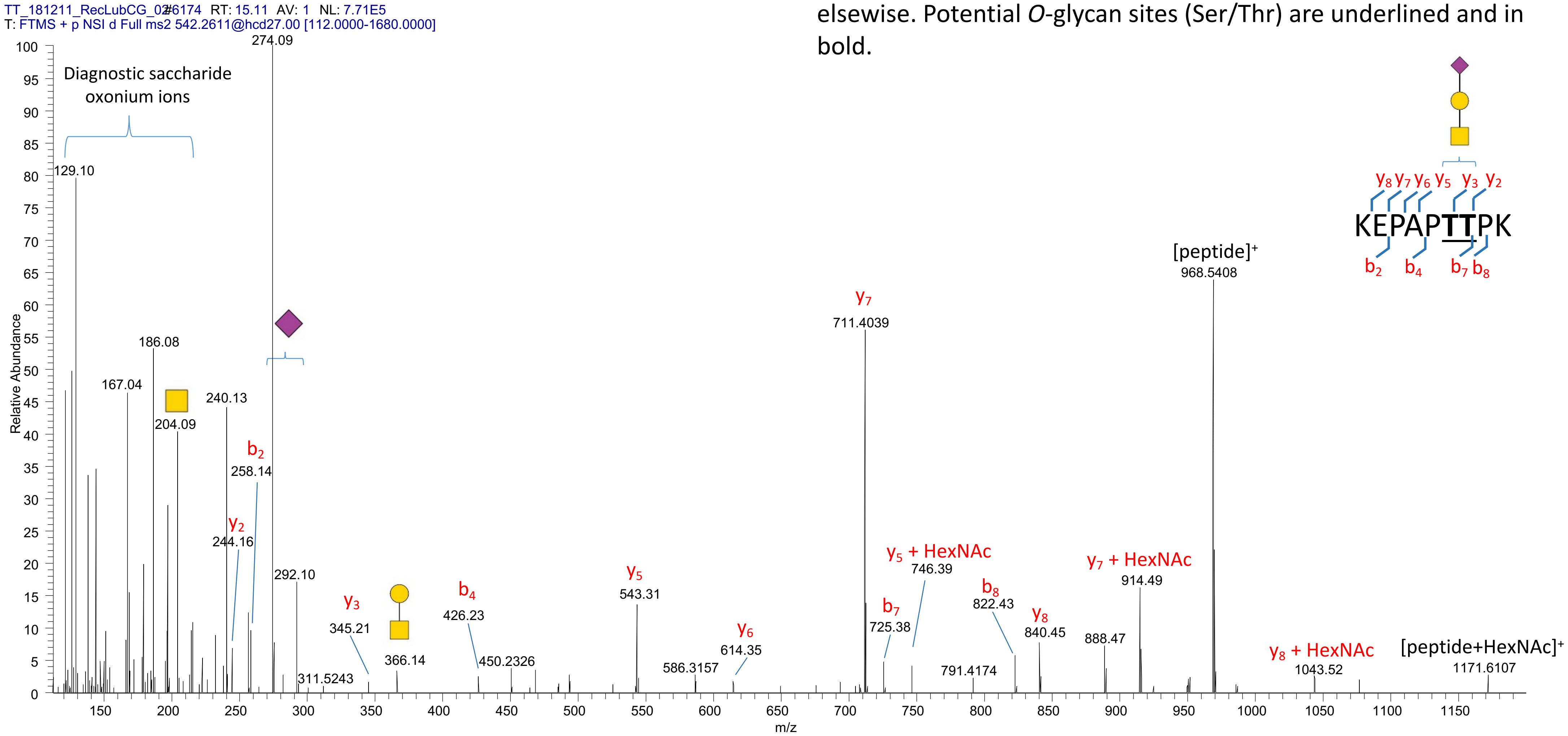

L.KEPAP**<u>TT</u>**PKKPAPK.E + HexNAc(2) Hex(2) NeuAc
(Amino acid position in sequence: 717-731,787-800)
Observed *m/z:* 837.7499 (3+)
Calc. $[M+H]^+$ = 2511.2335 , (Δppm= 0.68)

Supplementary Figure S10

HCD spectra of an *O*-glycopeptide from the mucin domain of recombinant lubricin (rhPRG4), digested with cathepsin G, and analysed with LC/MS/MS using a Qexactive instrument. Analytical conditions are described in Materials and Methods. Diagnostic glycan ions are detected in the lower mass range (*m/z* 100-400). b/y-ions are detected without glycan substituents if not annotated elsewise. *O*-glycan sites (Ser/Thr) are underlined and in bold.

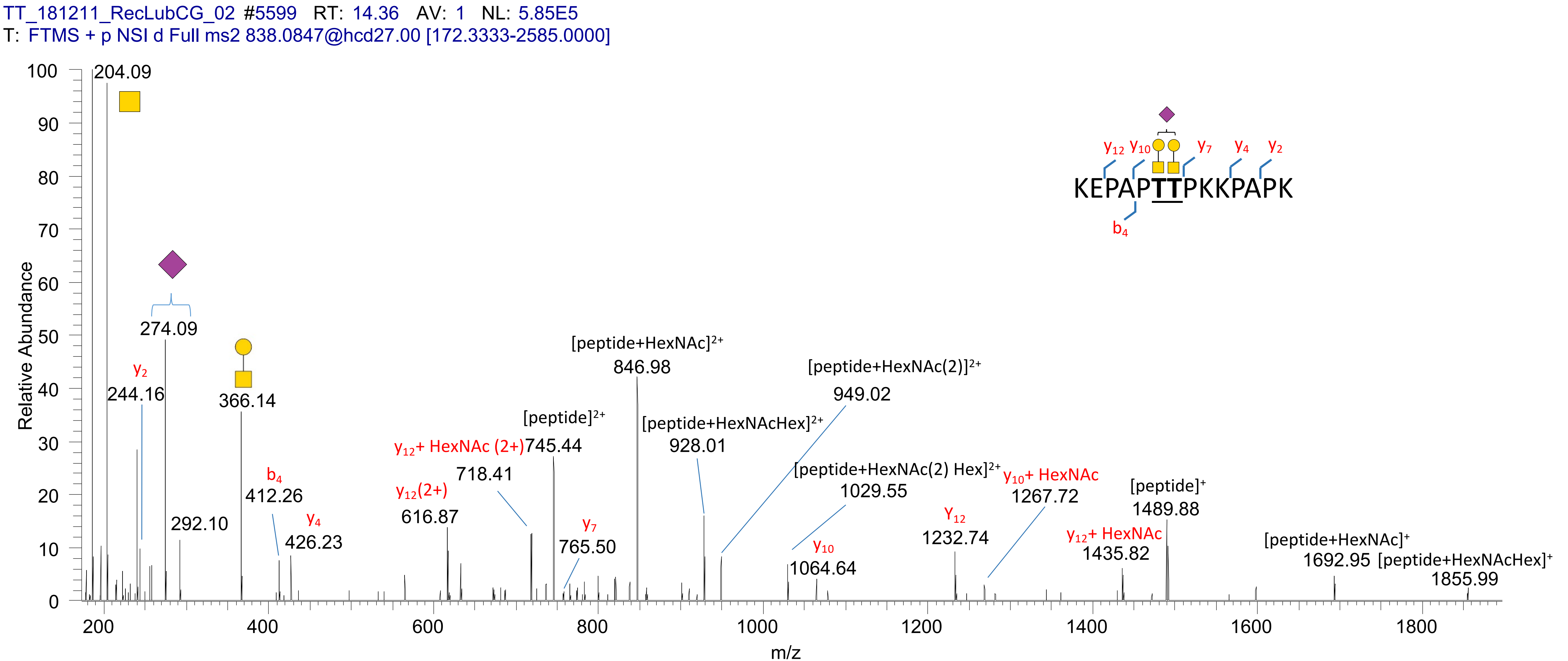

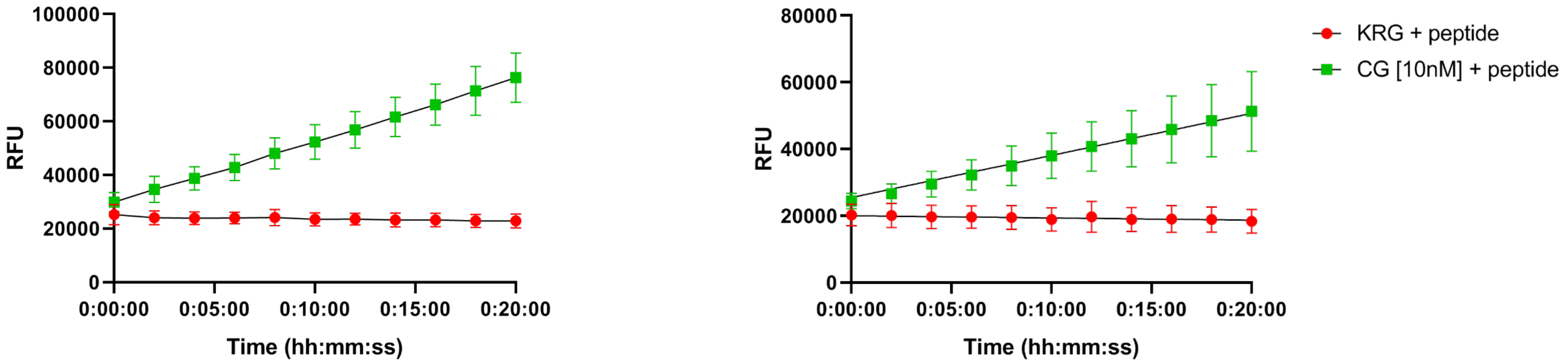

Supplementary Figure S10. The activity of cathepsin G (CG) used for the experiments in this project was assessed in a digestion assay using the fluorescent peptide 'Abz-EPFWEDQ-EDDnp' in Krebs-Ringer bicarbonate buffer (KRG). The assay was performed at two different occasions, and performed in triplicates, where mean +/- SEM are shown. See materials and methods for details.
